# Dynamic assessment of a humanized bone tumour microenvironment reveals insights into osteosarcoma primary tumour remodelling and lung metastases

**DOI:** 10.1038/s41598-025-29941-z

**Published:** 2025-11-28

**Authors:** Jonathan Gospos, Markus Laubach, Flavia Medeiros Savi, Akhilandeshwari Ravichandran, Julian Bauer, Oliver Friedrich, Boris M. Holzapfel, Ferdinand Wagner, Tomoji Mashimo, Siamak Saifzadeh, Dietmar W. Hutmacher, Jacqui A. McGovern

**Affiliations:** 1 Max Planck Queensland Centre (MPQC) for the Materials Science of Extracellular Matrices, Queensland University of Technology (QUT), Brisbane, QLD 4000 Australia; 2 Centre for Biomedical Technologies, School of Mechanical, Medical and Process Engineering, Queensland University of Technology, Brisbane, QLD 4000 Australia; 3 Australian Research Council (ARC) Training Centre for Multiscale 3D Imaging, Modelling, and Manufacturing (M3D Innovation), Queensland University of Technology, Brisbane, QLD 4000 Australia; 4 Department of Orthopaedics and Trauma Surgery, Musculoskeletal University Center Munich (MUM), University Hospital, Ludwig Maximilian University (LMU) Munich, Munich, Germany; 5 Department of Pediatric Surgery, Dr. von Hauner Children’s Hospital, LMU Munich, Munich, Germany; 6 Pediatric Clinic of Orthopaedics, Behandlungszentrum Aschau GmbH, Chiemgau, Germany; 7 Division of Animal Genetics, Laboratory Animal Research Centre, Institute of Medical Science, The University of Tokyo, Tokyo, Japan; 8 Division of Genome Engineering, Center for Experimental Medicine and Systems Biology, Institute of Medical Science, University of Tokyo, Tokyo, Japan; 9 Medical Engineering Research Facility, Queensland University of Technology, Chermside, QLD 4032 Australia; 10 ARC Training Centre for Cell and Tissue Engineering Technologies, Queensland University of Technology, Brisbane, QLD 4000 Australia; 11 Translational Research Institute, School of Biomedical Sciences, QUT, Woolloongabba, Brisbane, 4102 Australia; 12 Institute of Medical Biotechnology, Department Chemical and Biological Engineering, Friedrich-Alexander University Erlangen-Nürnberg, Erlangen, Germany

**Keywords:** Cancer, Oncology

## Abstract

Osteosarcoma (OS) is the most common primary bone cancer in children and adolescents, with poor prognosis linked to metastasis and recurrence. Preclinical models that replicate human disease progression and the tumour microenvironment (TME) are critical for advancing therapies. We developed a novel orthotopic and humanised bone tumour microenvironment model in highly immunocompromised Il2rg and Rag2 double knockout rats. A humanised bone niche (HN) was established by implanting orthotopic humanised bone constructs (ohTEBCs) around the rat femur, followed by SaOS-2-luc cell injection. Longitudinal µCT and bioluminescent imaging tracked HN formation, tumour growth, and metastasis. Histological and immunohistochemical analyses revealed pathological mineralisation, extensive collagen deposition, tumour-specific ECM production, and neovascularisation within primary tumours and lung metastases. This model supports consistent engraftment of SaOS-2-luc tumours with robust metastatic spread, recapitulating key human OS features. It offers a translatable preclinical platform for studying OS progression and testing chemotherapy and surgical interventions.

**Supplementary Information:**

The online version contains supplementary material available at 10.1038/s41598-025-29941-z.

## Introduction

Osteosarcoma (OS) is the most common primary malignant bone tumour in humans and is characterised by the formation of osteoid or immature bone caused by malignant cancer cells^1–4^. The pathogenesis and aetiology of OS is still unclear, although several risk factors have been identified such as genetics, prior chemotherapy, irradiation, Paget’s disease in the adult population and other benign bone lesions^5–7^. At the diagnosis of high-grade OS, less than 20% of patients have metastatic spread, however, this poses a significant additional lethal threat to the affected patients^8,9^. OS metastasis more often occurs in the lungs, and less commonly in bones with very uncommon spread to regional lymph nodes^8,9^. OS has a bimodal age distribution affecting adult populations over the age of 65 years and early adolescence’s where it is most prevalent^10^. In this adolescent population demographic, OS occurs commonly at the metaphysis of long bones, such as the distal femur, the proximal tibia or humerus^11,12^. Currently, OS is treated with a combination of systemic chemotherapy and surgical resection of the tumour. Poor disease outcomes and local recurrence remain a problem for OS patients. Over the past 40–50 years, improvements in the 5-year overall survival and recurrence rates of OS have been stagnant at approximately 60% and 10%, respectively^1^. This plateau suggests that current therapeutic strategies may not fully address the biological complexity of OS progression, including dynamic interactions between tumour cells and the surrounding microenvironment. Greater insight into these interactions is essential to guide the development of more effective therapies.

Understanding disease progression, developing new treatment options, and improving current therapeutic interventions for OS requires robust preclinical models that are permissible to human xenografting. Ideal models must also accurately recapitulate OS disease processes including primary tumour formation and metastasis to the lungs, allowing for the study of surgical interventions in addition to chemotherapy treatment. Highly immunocompromised animals (mice) have led to improvements in xenografting capabilities due to the impaired host immune system. More recently, humanization of the tumour microenvironment (TME) through harnessing tissue engineering and regenerative medicine (TE&RM) principles has allowed further improvements to mouse xenograft models. This refinement has led to improved engraftment rates and the improved ability to further study biologically relevant disease processes^13–15^. For example, SaOS-2 osteosarcoma cells grown within a humanized bone niche (HN) exhibit morphology and behaviour more consistent with patient-derived tumours than those grown in non-humanized site^16–21^. Such models offer a powerful platform to investigate dynamic TME remodelling, which is increasingly recognized as a driver of OS progression, treatment resistance and metastasis.

However, to study long-term disease dynamics and complex treatment interventions, it is essential to use an animal that has a longer lifespan and larger body size compared to mice. The increased anatomical scale of rats allows for more accurate modelling of clinically relevant OS therapies, including surgical resection and both neoadjuvant and adjuvant chemotherapy. Rats therefore overcome many of the limitations of mouse models, offering improved translatability for assessing tumour growth, metastatic progression and therapeutic response^22^. Building on this, our team previously established orthotopic and humanized TME models of OS within an immunocompromised X-SCID (Il2rg knockout; KO) rat^23^. In this model, the primary tumour developed within the HN and spontaneous lung metastasis was observed in 75% of the rats by the experimental endpoint, recapitulating key features of human disease^23^. This platform now provides an opportunity to examine the dynamic remodelling of the tumour-stroma interface in a biologically relevant and scalable in vivo setting.

In this study, we hypothesized that the recent development of a highly immunocompromised Il2rg and Rag2 double KO rat on an F344 background would allow to achieve the greatest possible efficiency in recreating an OS model with improved humanization rates and metastasis outcomes. The purpose of this study was to^1^ establish a permissive HN at the rat femur to support orthotopic OS growth within a relevant humanized microenvironment^2^, establish a humanized TME by orthotopically injecting the SaOS2-luc cell line into the HN to form a primary OS tumour^3^, perform longitudinal and dynamic in vivo assessment using µCT and bioluminescence imaging (BLI) to evaluate primary OS tumour development and lung metastasis, and^4^ perform detailed histological and immunohistochemical analyses to examine TME features of the OS tumour. Ultimately, these results support that OS development in a humanized bone niche rat model recapitulates key features of human OS disease, including bone remodelling, ECM deposition and lung metastasis.

## Materials and methods

### Medical–grade polycaprolactone (mPCL) scaffold preparation

Melt electro-written (MEW) tubular mPCL scaffolds with a 6 mm length and 5 mm diameter were fabricated as previously described (Supplementary Fig. 1)^24,25^. Plasma treatment (Harrick Plasma Cleaner PDC-002-HP) was performed with oxygen and argon for five min prior to coating with calcium phosphate (CaP) using a previously optimised protocol^26,27^. Briefly, plasma-treated mPCL scaffolds were submerged in 10X Simulated Body Fluid (SBF 10X), vacuum treated to remove air bubbles and incubated at 37 °C for 30 min. This process was repeated twice. The SBF 10X consisted of 1 M NaCl (ChemSupply, Gillman, SA, Australia), 5mM KCl, 25 mM CaCl_2_.2H_2_O, 5 mM MgCl_2_.6H_2_O (all from Merck, Darmstadt, Germany) and 10 mM Na_2_HPO_4_ (Ajax Finechem, Taren Point, NSW, Australia). Scaffolds were next placed in 0.5 M NaOH for 30 min at 37 °C, then rinsed at least five times with distilled water (ddH_2_O) to ensure the removal of residual NaOH. Prior to cell seeding, the scaffolds were sterilised by submersion in 80% (v/v) ethanol for 15 min before allowing to air dry in a tissue culture hood overnight.

#### Orthotopic and humanized bone construct (ohTEBC) creation

Human osteoblasts (hOBs) were isolated from bone explants from a female undergoing hip arthroplasty surgery following informed consent (using experimental protocols and regulations approved by the QUT Human Research Ethics Committee, approval no. 1400001024). All human cell work followed the National Health and Medical Research Council (NHMRC) National Statement on Ethical Conduct in Human Research 2023. Female hOB were used to match the female rats and female origin of the SaOS-2 cell line. A single hOB donor was used for these experiments and was selected based on pre-screening for high osteogenic potential using in vitro matrix mineralization assessment. The hOBs were expanded in growth media (MEM Alpha media, 10% foetal calf serum (FCS), 100 µg/mL streptomycin, 100 IU/mL penicillin (all from Gibco, ThermoFisher)) for two passages until 70–80% confluent, before seeding at 4 × 10^5^ cells per 35µL of serum-free growth media per scaffold^24^. After 2 h the hOB-seeded scaffolds were completely covered with growth media and cultured for two weeks before being switched to osteogenic media for a further four weeks (growth media supplemented with 10 mM β-Glycerophosphate, 50 µg/mL Ascorbate-2-phosphate, and 100 nM Dexamethasone (all from Sigma-Aldrich)). Six weeks of total in vitro pre-culture was allowed for the hOBs to seed and interconnect onto the mPCL scaffold and images of the in vitro culture were captured using a Nikon Ts2 brightfield microscope (Supplementary Fig. 1b).

### In vitro validation of the ohTEBC

#### Live/dead imaging

To assess cellular viability, live/dead staining was performed at weeks 2 and 6 after seeding hOBs onto the mPCL-CaP scaffolds (Supplementary Fig. 1c). At each time point, the ohTEBC was washed in phosphate buffered saline (PBS), then incubated in PBS containing 10 µg/mL fluorescein diacetate (FDA) and 5 µg/mL propidium iodide (PI) (both from Invitrogen™, Thermo Fisher Scientific) for 15 min protected from light, before transfer to fresh PBS prior to imaging^28^. The ohTEBC was imaged using a Leica SP5 confocal scanning laser microscope (Leica Microsystems GmbH, Wetzlar, Germany).

#### Immunofluorescence (IF)

Immunofluorescence (IF) was performed on ohTEBC samples collected following 2, 4, and 6 weeks in vitro culture to assess the maturation of the mineralized matrix and bone extracellular matrix (ECM). The ohTEBC samples were fixed in 4% Paraformaldehyde (PFA) for 60 min at room temperature (RT) and stored in PBS until IF was performed. Samples were permeabilised with 0.2% Triton X-100 in PBS containing 2% bovine serum albumin (BSA) for 10 min at RT, before washing twice with 1% BSA in PBS. Blocking was performed with 2% BSA in PBS for 10 min at RT before staining in a 1% BSA in PBS solution containing primary antibodies against Osteonectin, Osteopontin (both from DSHB), or Osteocalcin antibody (Abcam) at 4 °C overnight (each 1:100 dilution). The samples were then washed and incubated with 1% BSA in PBS containing 4’,6-diamidino-2-phenylindole (1 µg/mL; DAPI), Alexa Fluor 633 Phalloidin (1:300; Life Technologies), and Alexa Fluor 488 conjugated goat anti-rabbit (RRID: AB_143165) or anti-mouse (RRID: AB_2534069) secondary antibody (Life Technologies). IF-stained samples were imaged using a Leica SP5 confocal scanning laser microscope (Supplementary Fig. 1d). Mean fluorescence intensity was measured in Fiji (ImageJ) via splitting the colour channel into green and measuring the mean grey level on an 8-bit grey scale between 0 and 255. A total of three regions of interest were selected for analysis in each image (Supplementary Fig. 1e).

#### Scanning electron microscopy (SEM)

Scanning electron microscopy (SEM) imaging was used to visualise the structural details of the ohTEBC after 2, 4 and 6 weeks in culture. Scaffold samples were fixed in 2.5% Glutaraldehyde in PBS at RT for 1 h, then rinsed and stored in PBS. Dehydration of the sample was performed using graded ethanol concentrations of 10%, 20%, 30%, 40%, 50%, 60% 70%, 80%, 90% (twice) and 100% (twice). At each dehydration step, a 250-Watt Pelco Biowave (microwave) was used for 40 s with no vacuum. Critical drying was performed two times using 100% hexamethyldisilazane (HMDS) and utilising the Pelco Biowave (250-Watt) for one min with no vacuum. Samples were imaged using the Phenom XL G2 Desktop SEM (Supplementary Fig. 1e).

### In vivo experiment

#### Animal housing and handling

Fifteen female immunocompromised F344 Rag2/IL2rg double knockout rats were bred at the Pharmacy Australia Centre for Excellence (PACE) and housed at the Translational Research Institute (TRI), University of Queensland Biological Resource Facility (UQBR). In vivo breeding colony maintenance and experimental procedures were approved by the University of Queensland Animal Ethics Committee (following methods in approval numbers 2021/AE000227 and 2021/AE000240, respectively). All animal experiments in this study were conducted in accordance with the guidelines of the Australian Code of Practice for the Care and Use of Animals for Scientific Purposes, and the Animal Research: Reporting of In Vivo Experiments (ARRIVE 2.0) guidelines^29^. Rats were aged between 18 and 22 weeks prior to in vivo experimentations and were on a 12 h light cycle. They were provided *ad libitum* access to food and water and bedding was changed twice weekly. All rats were routinely monitored using a welfare score sheet and were of good health and maintained weight for the duration of the study (Supplementary Fig. 2).

#### Orthotopic ohTEBC implantation surgery

The rats weighed between 180 and 210 g at the time of ohTEBC implantation surgery. Rats were anaesthetized using 3% isoflurane in the induction chamber and transferred to a nose cone in a biosafety cabinet for anaesthesia maintenance. Isoflurane was adjusted between 1 and 2% to maintain surgical plane of anaesthesia, which was confirmed by absence of righting reflex, and toe and skin pinch. Buprenorphine (0.05 mg/kg) was administered via subcutaneous injection for pain relief 30 min prior to surgery. Once the rats were confirmed to be in the deep plane of anaesthesia, eye lubricant was applied, and the rat’s nose was secured within the isoflurane nose cone. The rat was placed on the heating mat in a left lateral decubitus position and the right upper thigh was completely clipped. Betadine was applied to the surgical area prior to incision. Sterile surgical instruments were utilised for interventions.

The right femur was palpated from proximal femur to patellar ligament to locate the isthmus of the femur in a lateral position. With a 15-blade surgical scalpel, a longitudinal incision was made laterally extending 20 mm from the inferior border of the greater trochanter and continued to the distal end of femur. The subcutaneous fat and muscle fascia were split in line with the skin incision. A pair of Mosquito forceps were used to separate the muscle fibres of *vastus lateralis* and *biceps femoris* and the tip was used to locate the femur. The forceps were expanded to expose the proximal femur. The process was continued to expose the distal femur for appropriate access to implant the ohTEBC. Once the femur was exposed, the ohTEBC was prepared. 30 µL of recombinant human bone morphogenic protein (rhBMP)−2 (1.5 µg/µL; Infuse™ Bone Graft kit, Medtronic) was added to 50 µL of thrombin and pipetted between the scaffold, 50 µL fibrinogen (both TISSEEL fibrin sealant, Baxter Healthcare) was then added onto the ohTEBC and allowed to polymerise for 60 s. The scaffold was then cut longitudinally to wrap around the femur. Two tweezers were utilised to grasp the edges of the scaffold making sure that scaffold fibres were not damaged. The ohTEBC was wrapped around the femur and the fibrin glue matrix polymerized after two min allowing the scaffold to seal to the rat femur. Muscle fibres were replaced over the femur, and the surgical wound was sutured in layers using resorbable sutures (Ethicon Vicryl^®^, Johnson and Johnson). Rats were monitored post-surgery and were administered buprenorphine every 6–12 h for the next 48 h.

### Orthotopic injection of the SaOS2-luc cell line into the ohTEBC

#### SaOS2-luc cell culture

SaOS-2 osteosarcoma cells (ATCC HTB-85) originally derived from an 11-year old female, were transduced to express luciferase and were cultured in McCoy’s 5 A Modified Medium containing 10% FCS, 100 µg/mL streptomycin, 100 IU/mL penicillin (all from ThermoFisher) and 5 µg/mL Blasticidin S hydrochloride (Abcam)^21,30^. Media was changed twice per week and the cells were passaged once they reached 80–90% confluency. Prior to experimentation, the cells were validated by short tandem repeat profiling and were confirmed to be mycoplasma negative.

#### Orthotopic injection of human OS cells

Eight rats were continued to the next phase of experimentation. An orthotopic OS tumour was created by injecting SaOS-2-luc cells into the established HN. All rats were anaesthetized with isoflurane as described earlier, the HN was pinched gently between the index finger and thumb and the site shaved and disinfected with 80% v/v ethanol. Using a 23 Gauge needle, the HN cortical shell was punctured, the needle was then advanced until it reached the outer cortex of the rat femur. Next, an insulin syringe containing 3.75 × 10^4^ SaOS-2-luc cells/µL in PBS was used to inject 20 µL of SaOS-2-luc cells (750,000 cells total) into the HN to generate the primary tumour.

#### Bioluminescence imaging (BLI)

Primary OS tumour growth and metastasis was monitored with weekly BLI using an IVIS Spectrum (Perkin Elmer, USA). Sterile D-Luciferin potassium salt (Promega; 15 mg/mL in PBS) was administered at 150 mg/kg to each rat via intraperitoneal injection under isoflurane anaesthesia. The rat’s right leg was shaved, and the rat placed into the IVIS in a left lateral decubitus position. Images were captured every min after injection until the tumour bioluminescence signal peaked. The rat’s lung area was shaved and then repositioned in a dorsal position to detect metastasis signal. On the scheduled endpoint day, the rats were injected with 150 mg/kg D-luciferin intraperitoneally approximately five min before CO_2_ asphyxiation and dissection. The organs, including lung, liver and leg were collected and ex vivo IVIS imaging was performed within 30 min. The resulting data were analysed using Living Image software v4.5.4 (Xenogen, CA, USA). The total flux in photons/second (p/s) within each defined region of interest (ROI) provided a surrogate of tumour burden.

#### Monitoring rats via in vivo micro–computerized tomography (µCT)

 In vivo µCT was performed to assess the creation of a HN and development of a primary OS tumour using a Molecubes X-Cube unit (Molecubes, Belgium). The scanning parameters were: 50 kV X-ray voltage, 350µA current, 960 exposures at 32 ms each, continuous single rotation, and binning factor of 1. Prior to OS tumour induction, µCT scans were performed at weeks 2, 4 and 6 weeks following ohTEBC implantation. Tumour growth was monitored via µCT at weeks 2, 4, 6, 8, 10, 12 and 14 after OS tumour induction. Images were reconstructed at 50 μm isotropic voxel size using Image Space Reconstruction Algorithm (ISRA). The bone volume and calcification of the HN and OS at the femur were quantified using VivoQuant software v2020 (Invicro, USA), and 3D surface rendering was accomplished using CTvox SkyScanner Software V3.3.1. For bone volume quantification, a region of interest was selected around the HN or OS tumour, and a Hounsfield Unit (HU) was set with a threshold between 400 and 6,000. The 3D volume of the HN and OS was then determined by subtracting the volume of the right femur from that of the left femur, as previously described from our team^23^. To align the femur position into true anatomical plane at the centre of the HN and OS, multiplanar reformats (MPR) was conducted and a set HU threshold was set between 400 and 6,000, using Materialise Interactive Medical Image Control System (MIMICS) Medical V24.0.0.

#### Euthanasia

Rats were euthanised by exposure to carbon dioxide (CO_2_) in accordance with the Australian Code for the Care and Use of Animals for Scientific Purposes. Animals were placed in a transparent chamber (Techniplast) connected to a regulated wall CO_2_ outlet. Gas was introduced at a flow rate of 20–40% of the chamber volume per minute until the chamber reached 100% CO_2_, where the animals remained for at least 5 min. Death was confirmed by the absence of respiration, heartbeat, and reflexes before tissue collection.

#### Ex vivo µCT

Fixed samples of the right femur, housing the HN and orthotopic OS tumour, along with the contralateral left femur, underwent ex vivo µCT. The Scanco Medical µCT 50 scanner (Scanco Medical AG, Brüttisellen, Switzerland) was used for high-resolution scanning with parameters set to a voxel size of 10.3 μm, a voltage of 55 kV, and intensity of 145 µA. The resulting data were then analysed using the Materialise Interactive Medical Image Control System (MIMICS) Medical V24.0.0.

Following ex vivo BLI, fixed lungs were dehydrated with a graded ethanol series (70%, 80%, 90% for 2 h each, then 100% ethanol for 8 h) followed by immersion in HMDS (Sigma-Aldrich) for 2 h and air dried overnight as previously described^31^. HMDS was used as a drying agent to preserve tissue architecture and enable high resolution µCT imaging of lung tissue, which otherwise lacks the inherent density contrast required for standard µCT. Dehydrated lungs were scanned using an ultra-high-resolution desktop µCT Scanner (Skyscan 1272; Bruker) with the following scan settings: 17 μm voxel size, 4 × 4 Binning, No Filter, 50 kV, 200 µA, 133 ms Exposure-time, Rotation steps = 0.3, Averaging = 4, Scan = 360°. After reconstruction (reconstruction details: (Reconstruction Software – Bruker-NRecon; Beam Hardening Correction – 20%; Smoothing – 0; Ring Artefact Correction – 10), 3D visualizations were created via Bruker-CTvox Software (Version 3.3.0).

#### Histology and immunohistochemistry (IHC)

After euthanasia and necropsy, all tissue samples were fixed in 4% PFA (Sigma-Aldrich) for seven days at 4 °C, before transfer and storage in 80% *v/v* ethanol at 4 °C until further processing. Prior to histological analysis, tissues were rapidly decalcified (KOS Multifunctional Microwave Tissue Processor, Milestone Medical, Italy) using 10% EDTA, pH 7.4 at 37 °C for 1 week (lung), up to seven weeks (right femur containing ohTEBC) or up to nine weeks (right femur containing ohTEBC and OS tumour), before processing (ASP300S Tissue Processor, Leica Biosystems, Germany) and paraffin embedding (Leica Histocore, Arcadia H, Leica Biosystems, Germany)). Serial paraffin Sect. (5 μm) were used for haematoxylin and eosin (H&E) staining and immunohistochemistry (IHC) as outlined in Supplementary Tables 1 and validated in Supplementary Fig. 3. All sections were deparaffinized via incubation in xylene followed by a graded ethanol series prior to rehydration in distilled H_2_O. Following antigen retrieval and washing with EnVision FLEX wash buffer (100 mL of wash buffer in 1,900 mL of water) (Agilent, REF: K800721-2), the tissues were delineated via DAKO pen (Agilent, REF: S2002). Endogenous peroxidase activity was quenched via incubation with 3% H_2_O_2_ in water (Sigma-Aldrich) for 5 min at RT followed by washing in wash buffer. Non-specific binding sites were blocked with 2% Bovine Serum Albumin (BSA) (Sigma-Aldrich, REF: A7906) for 30 min at RT followed by incubation with primary antibodies or isotype controls (Supplementary Table 1), followed by incubation in DAKO Envision + Dual link System HRP (Agilent, Ref: K406189-2) secondary antibody for 30 min. Positive immunoreactivity was detected using the DAB + 2-Component Immunohistochemistry Visualization System (Agilent; Ref: K346811-2). The slides were counterstained with Mayer’s haematoxylin prior to dehydration and mounting. IHC quantification of hsCol-I and CD68 was performed using Fiji (ImageJ) and the IHC toolbox plugin with a magnification factor of 10x. A total of three regions of interest were analysed within the primary tumour site. DAB and total neoplasm tissue area was converted to 8-bit binary, and a total threshold set at 243.

Tartrate-resistant acid phosphatase (TRAP) staining was performed on dewaxed and rehydrated paraffin section from the HN and OS tumour to assess osteoclast activity using a previously published method^32^. Briefly, the slides were incubated with 0.1 M acetate buffer (comprised of 0.1 M sodium acetate and 50 mM L-(+)-Tartaric acid (both Sigma-Aldrich) in ddH_2_O) for 20 min at RT. The slides were then incubated in 0.1 M acetate buffer containing 0.5 mg/mL naphthol AS-MX phosphate and 1.1 mg/mL Fast Red TR salt (both Sigma-Aldrich) for 45 min at 37 °C. Following incubation, the slides were washed in ddH_2_O, counterstained with Mayer’s haematoxylin (Sigma-Aldrich) and mounted with Aquatex (Aqueous mounting agent) (Merck).

#### Label–free multiphoton microscopy

To examine the interaction between the OS tumour and the adjacent muscular and connective tissue, thick primary OS tumour tissue sections at the rat femur (~ 1.5–2 mm) were optically cleared, using the PEGASOS clearing method which was formerly described by Jing, Zhang^33^, and subsequently analysed with multiphoton microscopy. The imaging was performed using an ultra-fast laser-scanning multiphoton microscope (TriMScope II, LaVision Biotech, Bielefeld, Germany) in combination with a mode-locked femtosecond-pulsed Ti: Sa laser (Chameleon Vision II, Coherent, Santa Clara, CA, United States). A 10x objective with a numerical aperture of 0.45 (CFI Plan Apochromat Lambda D 10x, Nikon Corporation, Tokyo, Japan) was used to focus the laser beam, which was tuned to a wavelength of 810 nm, with a pulse frequency of 80 MHz and an average output power of 60 mW. The emitted light, in backwards scattered direction, was collected by the excitation objective and subsequently detected by ultrasensitive transmission photomultiplier tubes (H 7422–40 LV 5 M, Hamamatsu Photonics, Herrsching, Germany). These were equipped with a 405/20 nm bandpass filter (CHROMA ET 405/20x, Chroma, Olching, Germany), to selectively detect Second-Harmonic-Generation (SHG) signal and a 525/50 nm bandpass filter, for tissue autofluorescence detection. The imaging parameters were adjusted to 1024 × 1024 pixels with an associated image size of 1092 × 1092 μm, which yielded a lateral pixel size of 1.07 μm and a pixel dwell time of 0.7 µs. To allow for the extraction of three-dimensional tissue morphometry parameters and to fully exploit the deep-tissue imaging capacities of this imaging modality, three-dimensional image stacks with depths of up to ~ 1.9 mm and a step-size of 1 μm, were recorded. In consideration of the lateral pixel size and the axial step size, a physical voxel size of 1.07 × 1.07 × 1 μm was achieved. Nonetheless, for demonstrative purposes, the images shown in Supplementary Fig. 6, only present a single image plane per specimen, to serve as an overview chart of the exact locations where the intensity profiles were determined. The profiles were created using the open-source image analysis software Fiji and were intended to provide deeper insight into potential collagen accumulations throughout the tissue section. However, it is to be mentioned that the polarization angle of the excitation light was not known during imaging and, therefore, polarization-dependent signal-intensity fluctuations cannot be ruled out.

#### vWF and red blood cell (RBC) quantification

Analysis of the vascular density in fixed lung tissue was performed by quantifying positive immunoreactivity of vWF on immunohistochemistry slides using Fiji (ImageJ) and the IHC toolbox plugin, magnification 5.0 ×^34^. DAB and the total lung tissue area was quantified after converting to 8-bit binary and selecting a threshold of 235. Three control slides were compared with three OS lung metastatic samples comparing a total of nine ROI.

Assessing RBCs in fixed H&E samples was completed using QuPath (Version 0.5.0) software. The ROI was selected at 40x magnification and haematoxylin separated. Cell detection and an optical density sum were performed to outline all cells in the field of view. Points annotation was then performed and assigned 10 positive RBCs and 10 negative cell nuclei. The cells were then put through an object classification or train object classifier to separate the cell types. Lung OS slides were compared to controls in the same method as vWF quantification.

### Statistical analysis

Statistical analysis was performed using GraphPad Prism (Version 10.4.1). Longitudinal data were analysed using a Friedman test with Dunn’s post hoc analysis. Comparisons between two groups was performed using paired and unpaired t-tests. Significance was assigned as follows: *P* ≤ 0.05 = *, *P* ≤ 0.01 = **, *P* ≤ 0.001 = *** and *P* ≤ 0.0001 = ****. Normal distribution of data was assessed by the Shapiro-Wilk test. Data were represented as bar graph (mean ± SD) or box plots, including median, first and third quartile, minimum and maximum, and were overlaid with individual data points.

## Results

### Humanized bone constructs form a permissive niche for orthotopic OS modelling

**Fig. 1:**
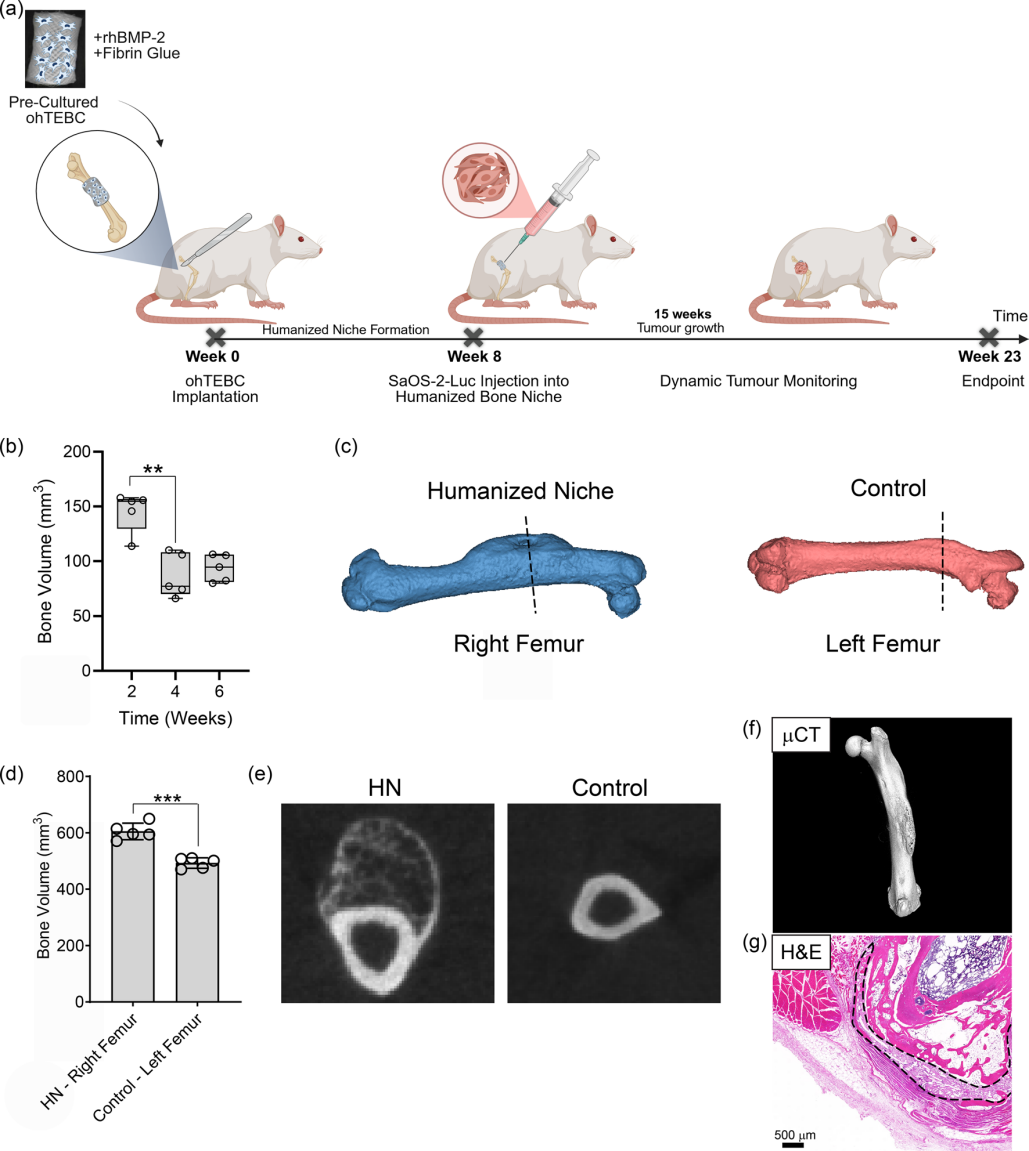
An ohTEBC forms a humanized bone niche in vivo. **(a)** Timeline of experimental procedure. The ohTEBC was precultured in vitro for 6-weeks prior to in vivo implantation. The ohTEBC was combined with rhBMP-2 and TISSEEL fibrin glue and implanted orthotopically around the right femur of F344 Il2rg Rag2 double knockout rats to form a human bone niche. The humanized bone niche matured over 8 weeks before SaOS-2-Luc cells were orthotopically injected into the microenvironment to form human OS at the primary site of the femur. The rats were dynamically monitored to characterize tumour development over a 15-week period. **(b)** Quantification of in vivo µCT scans showed an initial decrease in total bone volume (BV) followed by BV stabilisation at the right femur following ohTEBC implantation (*n* = 5). Friedman test revealed a significant effect of time on bone volume (*P* = 0.0085). Dunn’s multiple comparisons showed a significant decrease from week 2 to 4 (*P* = 0.0089), with no further significant change observed between weeks 4 to 6 (*P* = 0.1156). **(c)** Humanized bone niche (HN) formation was quantified using in vivo µCT scanning and reconstruction of the right femur vs. the contralateral left femur of the F344 Il2rg Rag2 Double KO rats (*n* = 5). **(d)** Bone volume analysis of µCT data after 6-weeks in vivo HN formation demonstrated a significant increase in bone volume within the right HN femur versus the contralateral left femur (*P* = 0.0003), displaying a mean difference of 112.1 ± 21.1 mm^3^ (mean ± SD, *n* = 5). **(e)** A 3D rendered axial (transverse) in vivo µCT slice of the HN at the (right) femur depicts a new cortical shell and trabecular bone after 6 weeks at the site of the surgically implanted ohTEBC, which is absent from the left contralateral femur. **(f**) High resolution µCT ex vivo 3D software reconstructions depict the human bone niche in continuum with the rats femur 10 weeks after ohTEBC implantation. **(g)** Cross-sectional H&E axial slice through the right femur demonstrating the morphology of the ohTEBC (dotted line) wrapped around the femur and the formation of an orthotopic HN. Statistical analysis comparing means was performed with a paired t test, **P ≤ 0.01, ***P ≤ 0.001. Created in part with BioRender. McGovern, J. (2025) https://BioRender.com/b5kqiwq

An in vivo humanized bone niche (HN) was created by implanting a pre-fabricated and pre-cultured ohTEBC around the rat femur of 15 immunocompromised F344 Ilr2g Rag2 double KO rats under sterile surgical procedure (Fig. 1a, Supplementary Fig. 1a-c). Quantitative analysis of the right femur total bone volume throughout the study revealed dynamic changes over time. Two weeks after the ohTEBC implantation, the average bone volume was 145.6 mm^3^ ± 18.4, decreasing to 86.7 mm^3^ ± 19.9 (−40.4%) by week 4, before slightly increasing to 93.7 mm^3^ ± 12.4 (+ 8.05%) at week 6. (Fig. 1b). Friedman test revealed a significant effect of time on bone volume (*P* = 0.0085). Dunn’s multiple comparisons showed a significant decrease in bone volume from week 2 to week 4 (*P* = 0.0089), with no further significant change between weeks 4 and 6 (*P* = 0.1156). This data suggests that the humanized bone may have been undergoing an active remodelling process to form the HN in vivo as has previously been observed in this model^13^.

Reconstruction of µCT scan images from the 6-week time point was performed to visualise and compare the calcified tissue volume between the right femur with the HN versus the contralateral left femur which did not receive an ohTEBC (Fig. 1c). The volume of the right femur (604.9 ± 29.5 mm^3^) was significantly larger (*P* = 0.0003) than the left femur (492.8 ± 18.6 mm^3^) (Fig. 1d). This demonstrated that the 22.75% increase in bone volume in the right femur compared to the contralateral left femur was attributable to the formation of a HN with a bone volume of 112.1 ± 21.1 mm^3^. Cross-sectional slices through the right and left femur demonstrate the HN formation compared to the control femur. The right femur included the presence of an extra-femoral cortical shell that seamlessly integrated into the rat femur and the presence of trabecular-like structure within the HN, which was absent from the left contralateral (control) femur (Fig. 1e). The presence of the HN was confirmed by performing high resolution ex vivo µCT (Fig. 1f) and subsequent morphological analysis (Fig. 1f) in a subset of rats. In H&E sections, the HN was wrapped around the rat femur, and fibrous-like tissue was present throughout the mPCL-CaP scaffold fibres. The HN contained a cortical shell and trabecular-like bone that integrated into the rat bone, and the rat femur remained intact and separated the femoral medullary canal from the HN. These data confirm that the implanted ohTEBC resulted in increased calcified tissue in the right femur which was due to the formation of an in vivo HN, distinct from the rat femur (Supplementary Fig. 4).

### The orthotopic humanized bone niche supports OS tumour growth and consistent lung metastasis

**Fig. 2:**
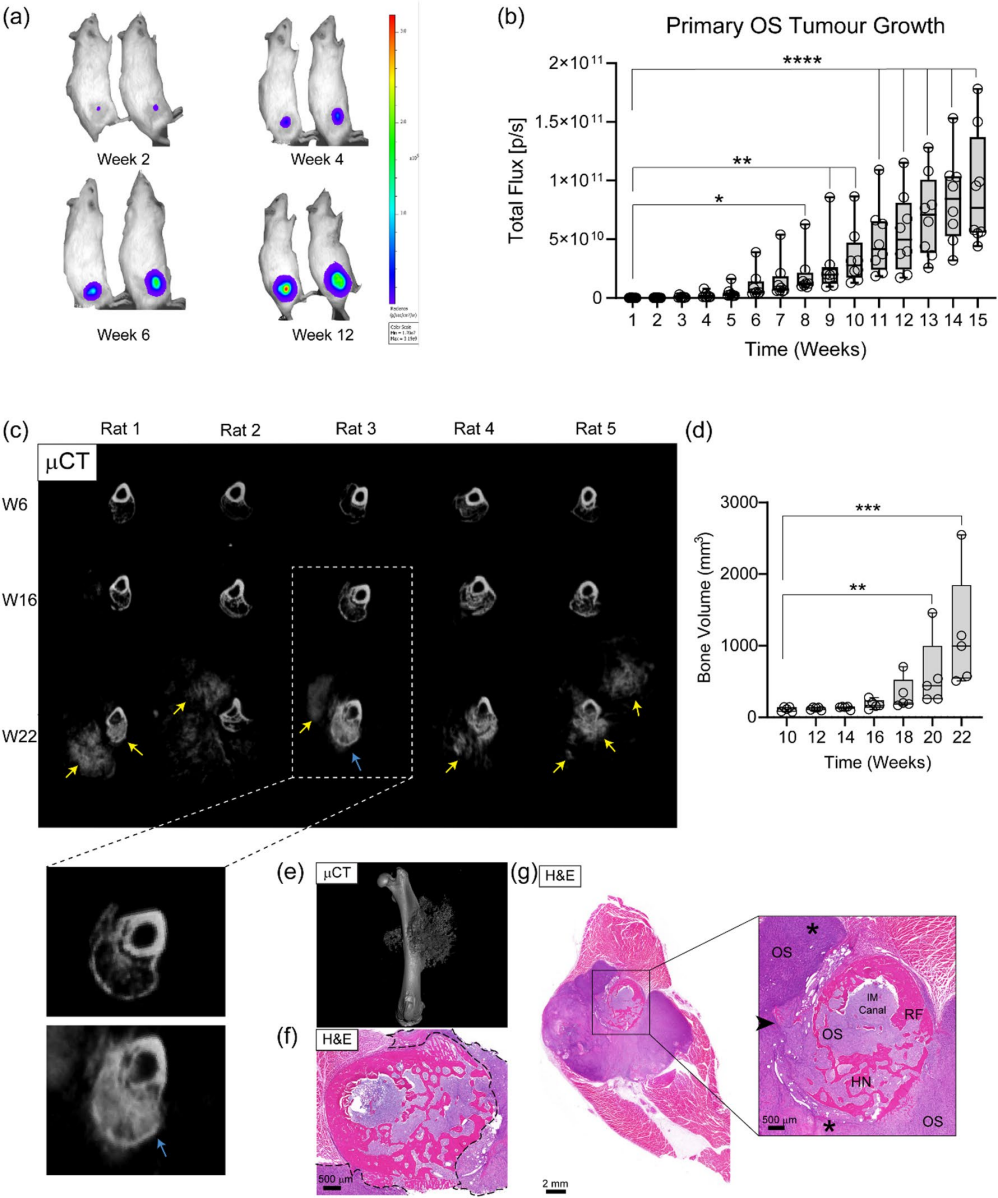
Longitudinal in vivo µCT and BLI imaging reveal dynamic osteosarcoma tumour growth and remodelling within the humanized bone niche. Rats (*n* = 8) were placed in a left lateral decubitus position to image the OS tumour growth over time. **(a)** Representative BLI images show the increasing SaOS-2-luc BLI signal at the right femur 2-, 4-, 6- and 12-weeks post-OS cell line injection. **(b)** Quantification of in vivo BLI signal over a 15-week period revealed a progressive increase in SaOS-2-luc tumour burden at the right femur following orthotopic injection of the OS cell line into the humanized niche (HN) (mean ± SD, *n* = 8). Friedman test revealed a significant effect of time on tumour growth (*P* < 0.0001). Dunn’s multiple comparisons test showed no significant difference in total flux compared to week 1 until week 8 (*P* = 0.0202), with stronger significance from Week 9 onwards (*P* < 0.01 to *P* < 0.0001). **(c)** Cross sectional µCT images of the right femur from individual rats 6-weeks after ohTEBC implantation demonstrating a new organised cortex forming the human bone niche (experimental week 6) (HN). Eight-weeks (experimental week 16) after orthotopic injection of SaOS-2-luc cells into the HN, the OS tumour was predominantly confined to the HN with thinning of the native rat femur cortex. By 14-weeks (experimental week 22) after induction of the orthotopic OS tumour, calcified tissue was detected outside the HN (yellow arrows), and the cortex of the rat femur appeared to be reabsorbed, and the OS tumour appeared to cause bulging on the HN (blue arrow and magnified insets). **(d)** An increase in total bone volume (BV) was confirmed via quantification of in vivo µCT scans. Friedman test revealed a significant effect of time on bone volume (*P* < 0.0001). Dunn’s multiple comparisons showed no significant change relative to week 10 until week 20 (*P* = 0.0077) and week 22 (*P* = 0.0005). Mean bone volume increased from 182.5 ± 62.1 mm^3^ at week 16 to 1154 ± 825.6 mm^3^ at week 22 (mean ± SD, *n* = 5). (**e**) High resolution µCT ex vivo 3D software reconstructions visualise the OS tumour expanding out from the HN at 15-weeks post orthotopic injection, which was confirmed by **(f)** Cross-sectional H&E axial slices demonstrating the OS tumour outside the HN (dotted lines). (**g**) A H&E cross-section of the right leg confirmed that the OS tumour had not invaded into the muscle tissue, but the HN (arrowhead) had been partially destroyed, and the OS tumour had invaded through the rat femur (RF) and into the intramedullary (IM) canal. The asterisks demonstrate the outward movement of mPCL-CaP struts away from the original HN location. W6, week 6, W16, week 16, W22, week 22. Values are shown as box plots with individual data points (*p ≤ 0.05, **p ≤ 0.01, ***p ≤ 0.001, ****p ≤ 0.0001).

Following confirmation of the formation of a HN in the F344 Il2rg Rag2 double KO rat model, an orthotopic OS tumour was induced within the HN of the eight remaining rats. The human SaOS-2-luc OS cell line was injected into the HN and monitored with BLI to track primary tumour growth. Imaging commenced one day after OS tumour induction and continued every week for 15-weeks in all eight animals. The rats were placed in a left lateral decubitus position to measure the orthotopic OS tumour (Fig. 2a), and the BLI signal remained as a confined tumour that continued to increase over time. Quantification of total flux revealed a significant effect of time on tumour growth (*P* < 0.0001). Dunn’s multiple comparisons post hoc analysis showed no significant change in BLI signal relative to week 1 until week 8 (*P* = 0.0202), with progressively stronger increases detected from week 9 onwards (*P* < 0.01 to *P* < 0.0001). At week 1, the mean BLI signal was 6.51 × 10^7^ ± 8.51 × 10^7^ p/s, increasing to 9.20 × 10^10^± 4.91 × 10^10^ p/s by week 15 (mean ± SD, *n* = 8) (Fig. 2b). In vivo and ex vivo BLI of the right femora confirmed that the OS tumour was present in all animals (Fig. 2b, Supplementary Fig. 5). All rats were monitored closely throughout the experiment for clinical wellbeing signs, and all were in good health throughout the experiment. These data confirm that the HN supports the growth of a primary OS tumour.

### Longitudinal imaging reveals dynamic OS tumour remodelling

Bioluminescent imaging confirmed the growth of the SaOS-2-luc cells within the HN. Continuous in vivo µCT was performed for five of the eight rats from the formation of the HN, induction of the OS tumour, until the experimental endpoint 15-weeks later (Fig. 2c). In all five rats, the HN was observed at the rat femur 6-weeks (w6) after implantation of the ohTEBC. µCT images taken 8-weeks after injection of the SaOS-2-luc cells (w16) show thinning of the rat femoral cortical bone at the interface between the HN, OS tumour and the endogenous rat bone. There is an increase in the appearance of trabecular-like structure within the HN and minimal presence of calcified tissue external to the HN. By 14-weeks post-OS cell injection (w22), the cortical bone at the junction between the HN, OS tumour and rat bone was absent in four of the five rats and severely thinned in one rat (Rat 2). This accounted for approximately 50% of the total rat femoral cortex. Interestingly, in Rat 3 the cortex of the HN appears to be bulging due to the growth of the OS tumour with evidence that the tumour had started to break out of the cortex into the extra scaffold area (Fig. 2c, blue arrow and higher magnification insets). In all rats analysed, the HN cortical shell was partially or completely degraded, and the trabecular-like calcified structures within the HN had been replaced with calcified tissue of sunburst appearance both within the HN and extending beyond the HN confines (Fig. 2c; yellow arrows and Supplementary Fig. 4).

Following injection of the SaOS-2-luc cells into the HN, there was a steady increase in bone volume at two weeks (110.8 mm^3^ ± 33.0) (18.2% increase), suggesting the development of an orthotopic OS tumour. At week 4 post OS induction (experimental week 12), there was an increase in bone volume by 8.30% (120.0 mm^3^ ± 20.9), this continued at week 6 (134.4 mm^3^ ± 22.4) with a 12% increase in bone volume. At weeks 8, 10, and 12 post-OS induction, there were steep increases of 35.8% (182.5 mm^3^ ± 62.1), 75.8% (320.8 mm^3^ ± 226.3) and 84.0% (589.5 mm^3^ ± 500.5) in bone volume, respectively. The trend continued with a final increase of 95.7% in bone volume at week 14 (1154.2 ± 825.6 mm^3^). Friedman test revealed a significant effect of time on bone volume (P = < 0.0001). Dunn’s multiple comparisons showed no significant differences in bone volume relative to week 10 until week 20 (*P* = 0.0077) and week 22 (*P* = 0.0005) (Fig. 2d). In vivo µCT data showed that after injecting the OS into the HN, the tumour growth was slow during experimental weeks 10–18, then significantly accelerated from weeks 20–22. This suggested that calcified tissue was forming within the OS tumour, contributing to the increase in bone volume measured within the HN.

Due to the rapid increase in the OS tumour size according to in vivo µCT, the rats were euthanized. High resolution ex vivo µCT was performed 15-weeks after OS cell injection on three representative right femora. The 3D reconstructions confirmed the expansion of calcified tissue from the HN where the OS cells had been injected and the typical sunburst appearance, a peculiar radiographic feature of fast-growing OS (Fig. 2e). The ex vivo right femurs were then processed for histology to further characterise the OS tumour and local tumour microenvironment. Morphological analysis with H&E staining confirmed the expansion of the primary OS tumour beyond the confines of the HN after orthotopic OS cell line induction (Fig. 2f). As suggested by BLI, the OS cells remained as a formed tumour that did not appear to invade into the surrounding muscle tissue (Fig. 2f), however, the high-resolution Second Harmonic Generation (SHG) imaging seems to suggest otherwise (Supplementary Fig. 6). The SHG results depict collagen-I fibres infiltrating between muscles at the OS/muscle interface (Supplementary Fig. 6). There was apparent destruction of the cortical bone of the rat femur intramedullary canal, as well as the cortical shell and trabecular bone within the HN (Fig. 2g). The results indicate that the OS tumour and HN undergo dynamic changes over time. This includes the destruction of the HN and rat femoral bone cortical shells, which aligns with significant growth of the OS tumour from experimental week 16, suggesting an OS-mediated destruction of the native bone microenvironment.

### Osteosarcoma actively remodels its local bone and tumour microenvironment

**Fig. 3:**
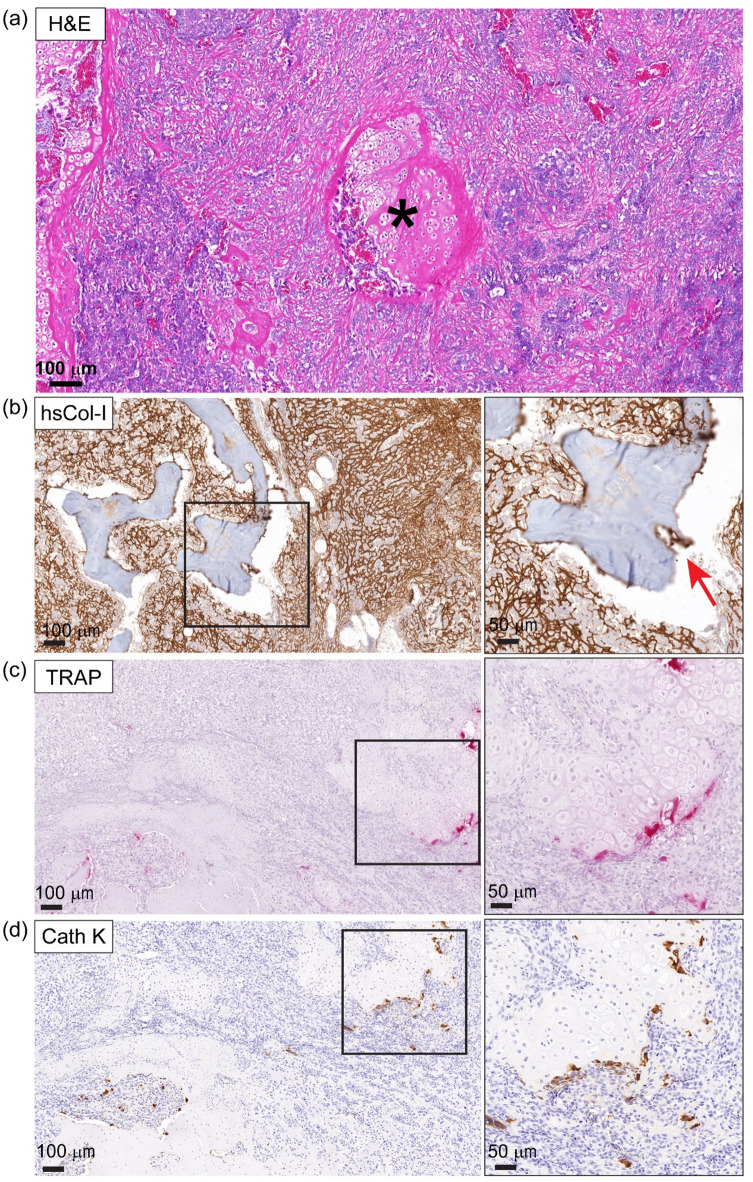
The bone and tumour microenvironments are actively remodelled during OS development. **(a)** H&E of primary OS demonstrating zones where new bone is being laid. OS osteoid surrounds a zone of endochondral ossification (asterisk). The scale bar represents 100 μm. (**b**) hsCol-I IHC (brown staining) shows that the bone appears to have the characteristic osteoclast cutting cone formation suggesting that it is being reabsorbed by the OS (inset, red arrowhead). **(c)** TRAP (pink staining) positive revealed osteoclasts at the interface at the HN and within the main body of tumour. **(d)** Cathepsin K (brown staining) confirming bone remodelling is osteoclast mediated. The scale bars represent 100 μm and 50 μm.

To better understand the destructive remodelling within the HN and OS tumour, histological characterisation of the orthotopic OS tumours was performed. H&E revealed areas of osteoid production and new bone formation within the OS tumour (Fig. 3a, asterisk). Detection of an osteoclast cutting cone in hsCol-1-stained sections suggested that the destructive resorption of the calcified tissue was driven by enhanced osteoclastic activity within the OS tumour (Fig. 3b). Tartrate-resistant acid phosphatase (TRAP) staining and Cathepsin K IHC were then performed to determine the presence and activity of osteoclasts within the OS tumour microenvironment. Positive TRAP staining was observed at the bone-tumour interface (Fig. 3c) in addition to Cathepsin K positive cells in a serial histological section (Fig. 3d), confirming HN bone resorption and remodelling occurred in an osteoclast-mediated process.

### OS proliferation drives ECM deposition, remodelling and immune cell recruitment

**Fig. 4:**
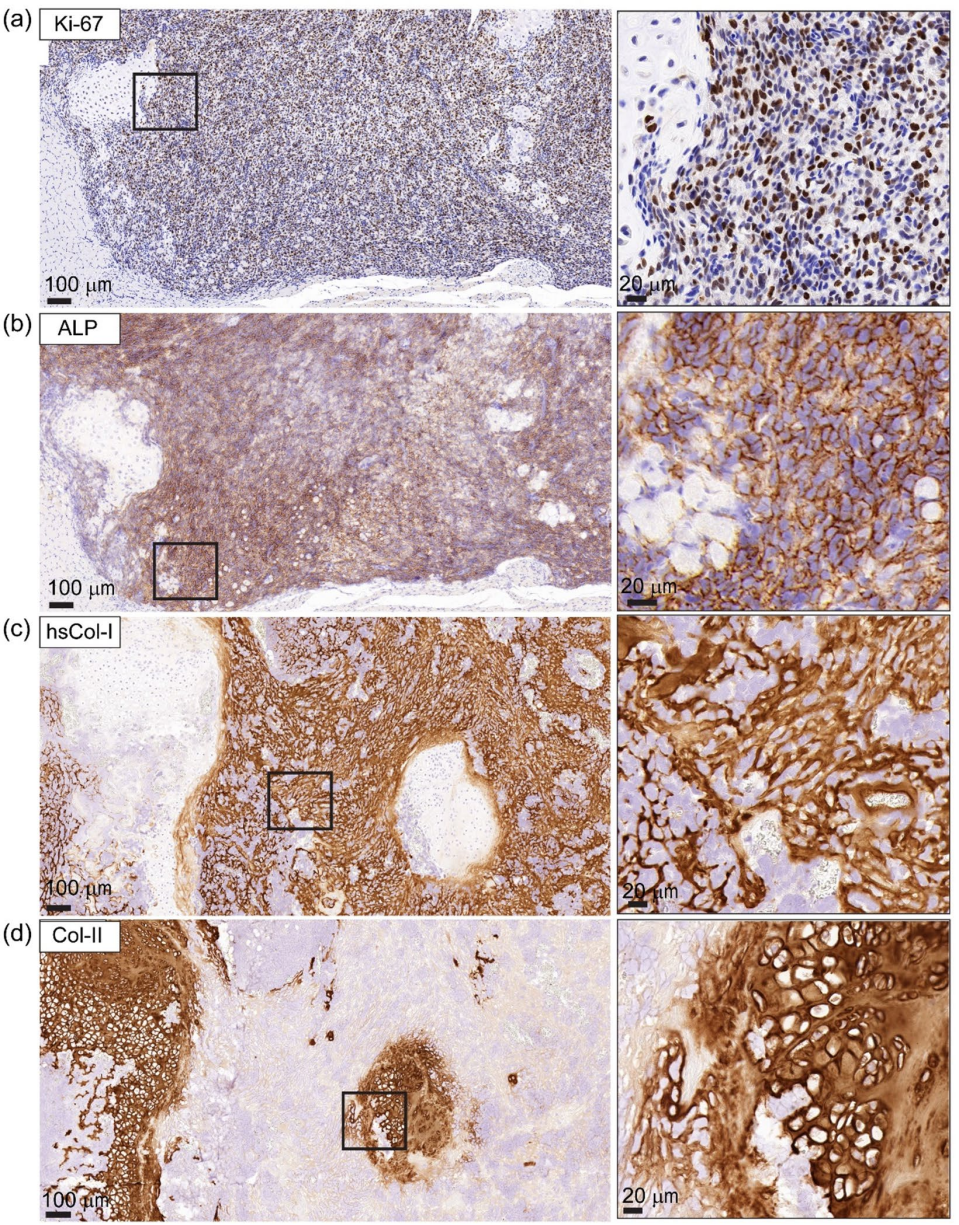
Osteosarcoma exhibits high proliferative activity and induces osteoid matrix deposition.**(a)** Ki-67 immunohistochemistry (IHC) reveals widespread nuclear staining in SaOS-2-Luc tumour cells, indicating a highly proliferative tumour. **(b)** Alkaline phosphatase (ALP) staining highlights areas of osteoblastic activity within the tumour, with a characteristic lace-like osteoid morphology. **(c)** Human-specific collagen type I (hsCol-I) IHC demonstrates extensive tumour-derived osteoid matrix deposition. **(d)** Collagen type II (Col-II) IHC reveals cartilage-like regions interspersed throughout the tumour, suggestive of endochondral ossification. Inset boxes show high magnification views of the IHC images. The scale bars represents 100 μm and 20 μm.

To investigate the drivers of rapid tumour expansion within the HN, we first evaluated proliferation and osteoblastic activity of the OS cells. IHC for proliferation marker, Ki-67 revealed that the OS tumour was highly proliferative, compared to the surrounding bone tissue (Fig. 4a). Alkaline phosphatase (ALP) is an indicator of osteoblastic activity and is highly expressed by OS cells (Fig. 4b), consistent with active osteoid production by the SaOS-2-luc cells. The OS tumour was characterised by a high cellular density, with extensive deposition of ECM throughout the tumour (Fig. 4c). Newly deposited ECM was predominantly hsCol-I (36.89 ± 7.48% of primary tumour was positive via IHC) (Supplementary Fig. 7a), the major component of OS osteoid ECM with a characteristic lace-like morphology. The OS tumour also appeared to create new ossification centres that had reabsorbed the femur and HN and surrounded the remaining viable bone (Fig. 4c, d). Further analysis showed islets of hypertrophic cartilage interspersed throughout the OS tumour microenvironment (Fig. 4d). The cartilage-like tissue was hsCol-I negative, but strongly positive for Col-II, suggesting endochondral ossification-mediated new bone formation or a mixed osteoblastic and chondroblastic OS tumour derived from the SaOS-2-luc cells.

**Fig. 5:**
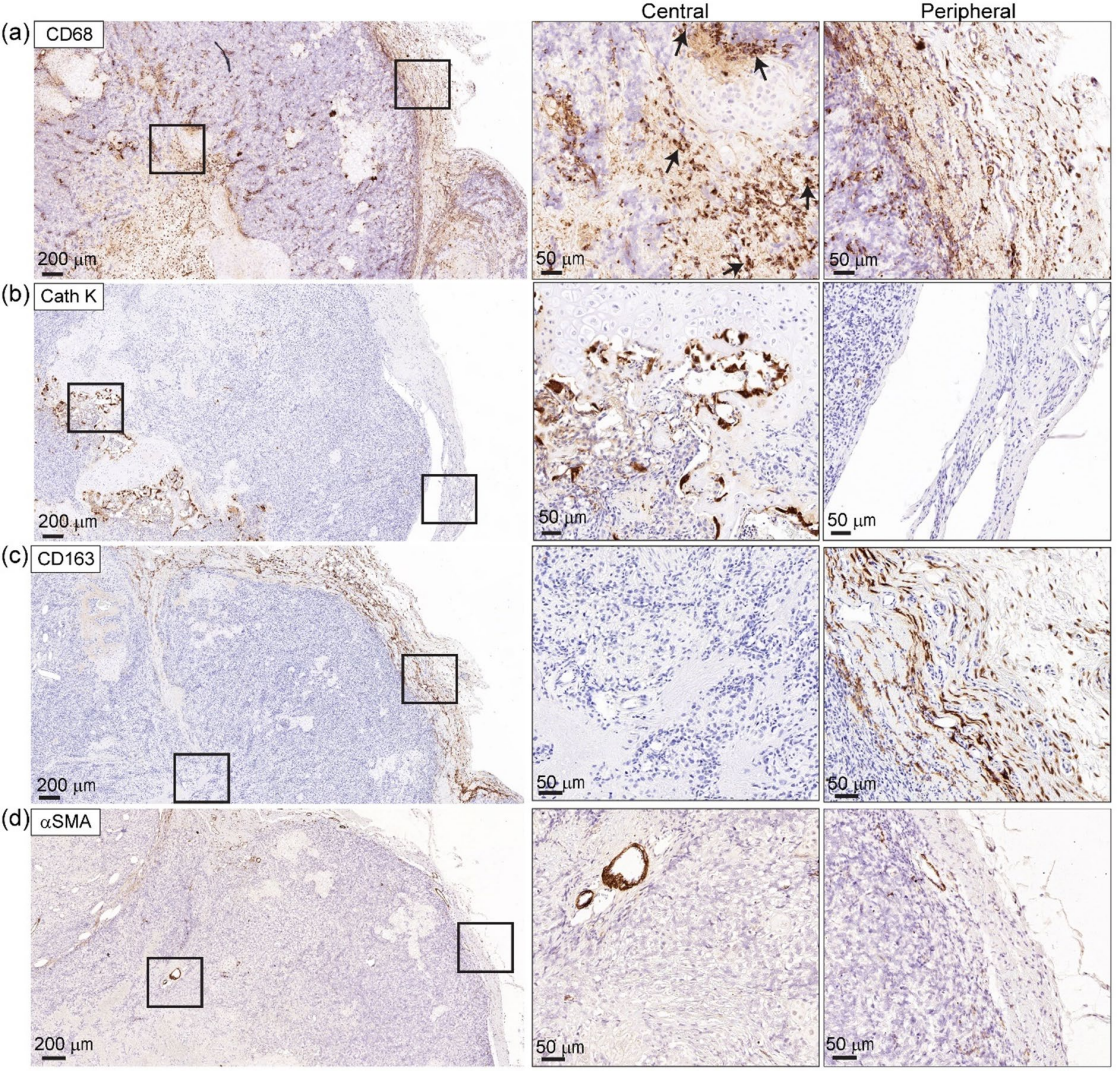
Osteosarcoma modifies immune cell recruitment and spatial macrophage polarisation. **(a)** CD68^+^ cells are found throughout the OS tumour microenvironment but are particularly enriched at the tumour periphery. Multinucleated CD68^+^ cells were present in the centre of the tumour (black arrows). **(b)** Cathepsin K (Cath K) demonstrates that osteoclasts are present in CD68^+^ regions of the tumour. (**c**) CD163^+^ M2-like macrophages are localised only to the outer margin of the tumour, suggesting spatially restricted polarisation. **(d)** αSMA staining is absent from the tumour boundary, indicating that exclusion of CD163^+^ cells is not due to a fibrotic capsule. Insets show higher magnification images of central and peripheral tumour regions. The scale bars represent 200 μm and 50 μm.

To further investigate the inflammatory response and bone destruction observed within the rat femur and HN, we assessed immune cell infiltration within the OS tumour. CD68 (3.42 ± 1.16% of primary tumour was positive via IHC) (Supplementary Fig. 7b) immunoreactivity identified the presence of cells from the monocyte lineage, suggesting an accumulation of macrophages and osteoclasts within the OS tumour, particularly at the tumour-bone interface (Fig. 5a, b). Interestingly, CD68^+^ cells had also accumulated around the periphery of the primary tumour and had a distinct morphology from the intratumour CD68^+^ cells (Fig. 5a), with smaller and more uniform cells typical of macrophages at the periphery and larger, multinucleated CD68^+^ cells present in the tumour centre with a phenotype more typical of osteoclasts (Fig. 5a). IHC with Cathepsin K confirmed presence of osteoclasts in the tumour centre versus the periphery (Fig. 5b). To further identify the monocytic cell types, CD163 IHC was performed to highlight the M2 polarized macrophage population. Intriguingly, CD163^+^ cells were concentrated only at the periphery of the primary tumour, suggesting that M2 macrophages were recruited to, but did not infiltrate the tumour (Fig. 5c). To determine whether this spatial restriction was due to the formation of a physical barrier, α-smooth muscle actin (αSMA) IHC was performed to detect the presence of a fibrous capsule. However, αSMA was not detected at the tumour boundary and was only present in vasculature surrounding the tumour (Fig. 5d), indicating that the exclusion of M2 macrophages from the central tumour was not due to fibrous capsule formation. Together, these findings demonstrate that the rapid expansion of the orthotopic OS tumour is driven by high cellular proliferation and deposition of osteoid ECM. The remodelling of the bone and tumour microenvironment is accompanied by macrophage recruitment and characterised by M2 polarized macrophage exclusion.

### OS lung metastases have pathological mineralization and secrete tumour–specific ECM

**Fig. 6:**
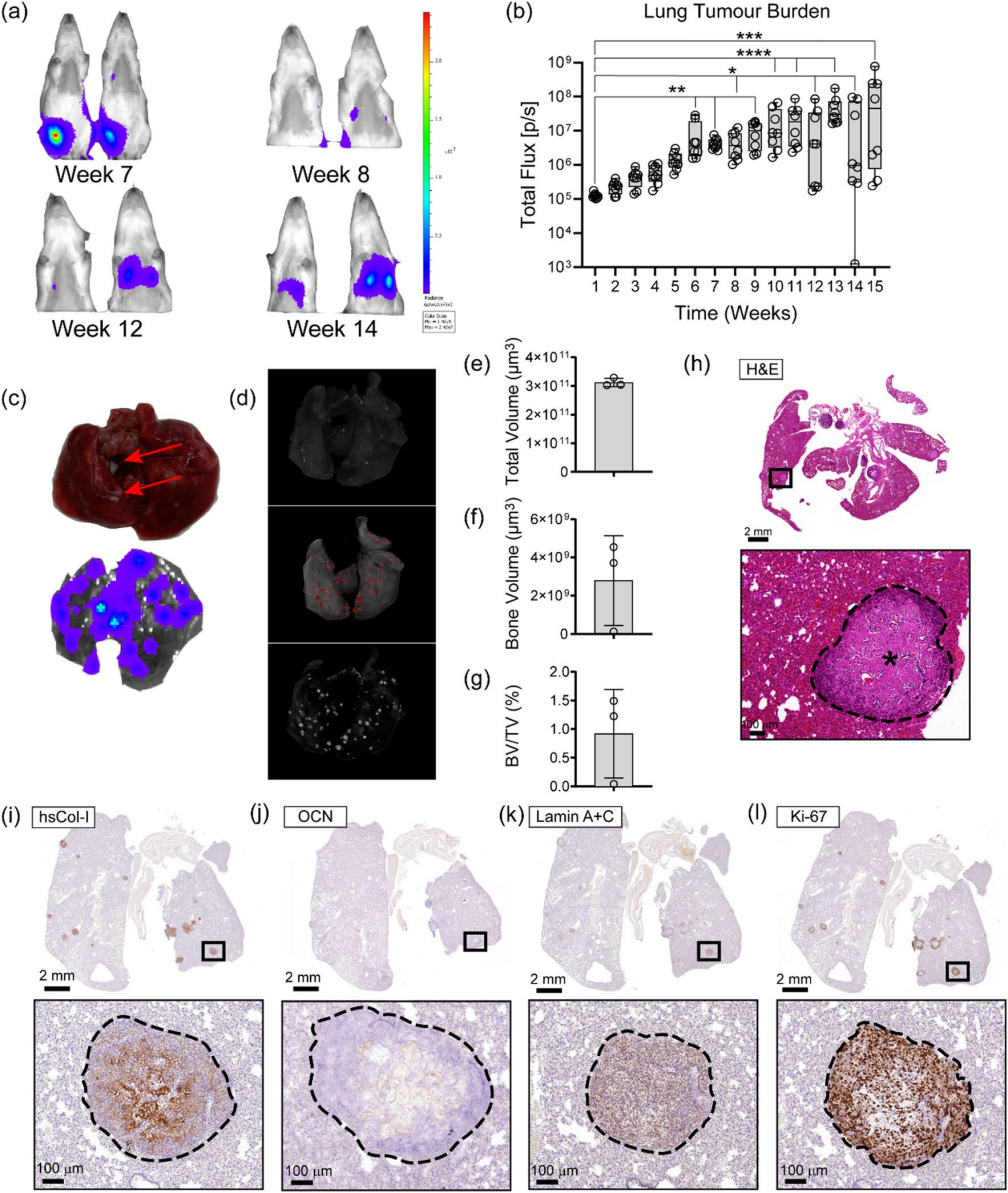
OS lung metastases form calcified nodules and secrete tumour-specific ECM. **(a)** Representative images show an increase in detectable BLI signal from the lungs over time. **(b)** Quantitative BLI signal analysis confirmed a progressive increase in lung tumour burden over 15 weeks (*n* = 8). Friedman test revealed a significant effect of time on lung tumour burden (*P* < 0.0001). Dunn’s multiple comparison test revealed no significant change relative to week 1 until significance was achieved at week 6 (*P* = 0.0018), with progressively stronger significance from week 7 onwards (*P* < 0.05 to *P* < 0.0001). **(c)** Representative macroscopic imaging of rat lung tissue with visible metastatic tumour nodules (arrows) and ex vivo BLI demonstrate positive flux signal confirming SaOS-2-luc-derived metastases. **(d)** µCT 3D reconstructs showed the 3D lung (with trachea and mediastinum). A windowed lung that revealed the lung nodules present (white nodules). Trachea and mediastinum ROI are cut out to display total volume of lung (grey) and a bone volume overlay is added to show lung nodules (red nodules) confirming OS metastasis from the primary tumour. **(e)** The total lung tissue and **(f)** bone volumes are calculated to represent a **(g)** BV/TV percentage of (0.89% ± 0.75% (mean ± SD, *n* = 3)) lung tissue occupied by calcified OS lung metastatic nodules as determined by quantitative µCT analysis **(h)** H & E staining demonstrated lung metastasis in a coronal view, and magnified view of the OS lung nodule (asterisk) confirmed the metastatic spread to the lungs from the orthotopic primary tumour. Representative cross-sectional coronal IHC of lung metastases depicted the deposition of a tumour-specific **(i)** hsCol-I ECM and **(j)** low expression levels of osteocalcin (OCN). (**k**) Positive Lamin A + C immunoreactivity confirmed the human-origin of the cells within the lung nodules and (**l**) Ki-67 confirmed the highly proliferative capacity of the metastases. Values are shown as bar graphs and box plots with individual data points (*P ≤ 0.05, **P ≤ 0.01, ***P ≤ 0.001, ****P ≤ 0.0001).

To evaluate whether primary OS tumours formed within the HN develop lung metastases, and to characterise their morphology and matrix composition, we used a combination of BLI, µCT, and histology. First, in vivo BLI was performed on the rats in dorsal position to monitor the metastasis of the SaOS-2-luc cells to the lungs (Fig. 6a). Friedmans test revealed a significant effect of time on lung tumour burden (*P* < 0.0001). Dunn’s multiple comparisons showed a significant increase in total lung flux relative to week 1 beginning at week 6 (*P* = 0.0018), continuing through to week 7 (*P* = 0.0035), week 8 (*P* = 0.0269), week 9 (*P* = 0.0011), week 10 and 11 (*P* < 0.0001), week 12 (*P* = 0.0222), week 13 (*P* < 0.0001), week 14 (*P* = 0.0324) and week 15 (*P* = 0.0003). At week 1, the total lung flux was 1.25 × 10^5^ ± 2.28 × 10^4^ p/s, increasing to 8.63 × 10^6^ ± 1.08 × 10^7^ at week 6, and reaching 1.69 × 10^8^ ± 2.67 × 10^8^ by week 15 (mean ± SD, *n* = 8) (Fig. 6b). Ex vivo lung BLI confirmed that 100% of the rats had developed lung metastasis (Supplementary Fig. 5). These data confirm that the HN supports the growth of a primary OS tumour and recapitulates human disease physiology by metastasis that consistently spreads to the lungs. Furthermore, we observed that colonisation of the lungs by SaOS-2-luc cells had occurred by 6-weeks post-OS cell line injection.

Following characterisation of the primary tumour, the macroscopic and microscopic features of the OS lung metastases were investigated. White nodules were visible on the surface of lung tissues upon necropsy in all animals, and these were confirmed to be OS lung metastases via ex vivo BLI (Fig. 6c). Ex vivo µCT performed on the rat lungs showed that the metastatic nodules had formed calcified tissue (Fig. 6d). The total lung tissue volume (TV) was 3.12 × 10^11^ ± 1.47 × 10^10^ µm^3^ (Fig. 6e), and the bone volume (BV) of the calcified OS nodules was 2.79 × 10^9^ ± 2.34 × 10^9^ µm^3^ (Fig. 6f), representing an average BV/TV of 0.89% ± 0.75%, or lung tissue occupied by calcified OS lung metastatic nodules as determined by quantitative µCT analysis (Fig. 6g). The OS lung metastases were confirmed with H&E staining which showed areas of pathologically distinct tissue. The periphery of the metastatic lung nodules was densely populated with cells with a lace-like osteoid ECM, surrounding a centre of sparsely populated irregularly shaped and interconnected osteoid islets (Fig. 6h). The central osteoid tissue exhibited strong staining for hsCol-I, while the lace-like peripheral osteoid tissue also showed positive hsCol-I staining, albeit with lower intensity (Fig. 6i). Low levels of weakly-staining OCN positive tissue in the centre lung metastatic ECM-network confirmed osteoblastic activity and potential immature bone formation (Fig. 6j). Lamin A + C showed that the lung metastases were of human origin, together with the BLI signal confirming that the SaOS-2-luc cells were the source of the metastases (Fig. 6k). Ki-67 IHC demonstrated that the tumour nodules were comprised of highly proliferative cells concentrated around the periphery and absent from the central space (Fig. 6l). These results demonstrated that proliferation was occurring outwards from the edges of the lung metastases, while ECM deposition and pathological calcified tissue formation was concentrated in the less proliferative central zone.

### OS metastases remodel the pulmonary vascular niche

Histology sections of lung tissue were further assessed to determine the changes in vascular density and architecture following the development of pulmonary OS metastases. Immunohistochemistry for von Willebrand Factor (vWF) was performed to assess vasculature in normal rat lung tissue in comparison to the rat lung tissue with metastasised OS tumours (Supplementary Fig. 8a-b). Quantification of vWF revealed a significantly lower (*P* = 0.0012) vWF surface area of 13.3% ± 2.87%, in normal lung tissue as compared to 17.85% ± 1.86% (mean ± SD, *n* = 9 ROI) for lung tissue with metastatic OS nodules (Supplementary Fig. 8c). To confirm the observed changes in vascular density, the area of lung tissue containing RBCs was quantified. Similar to vWF area, significantly lower (*P* = 0.0096) RBC area 60.88% ± 5.95% (mean ± SD, *n* = 9 ROI) within normal lung tissue in comparison to OS lung tissue 68.84% ± 5.52% (mean ± SD, *n* = 9 ROI) (Supplementary Fig. 8g). Together, this suggests that the presence of OS metastases in lung tissue is associated with an increase in vasculature density and contributes to the formation of an optimal metastatic niche to support metastatic growth.

## Discussion

In this study, we aimed to establish and characterize an orthotopic and humanized rat model of osteosarcoma (OS) by harnessing tissue engineering and regenerative medicine principles. Our approach to implant an orthotopic humanized bone construct (ohTEBC) around the rats’ femur led to the formation of a humanized bone niche (HN) that replicated the bone tumour microenvironment (TME). Subsequent intraosseous injection of SaOS-2-Luc cells into the HN enabled the development of primary OS tumours and consistent metastasis to the lungs. The highly immunocompromised Il2rg and Rag2 double knockout rat on an F344 background addressed the limitations of current mouse models, offering an in vivo model with a longer lifespan and larger body size, with comparable disease modelling capabilities^13,19–21^.

The local bone TME has an important role in supporting OS tumour growth and maintenance of disease phenotype, including lung metastatic behaviour^35^. Interaction of OS cells with a humanized bone TME further supports OS disease phenotype, including retention of novel marker expression^20^. Building upon previous work from our team^23^, we created a HN at the femur of highly immunocompromised F344 double knockout rats that supported OS growth and lung metastasis in 100% of the rats from this study. Dynamic and longitudinal in vivo µCT analysis of the HN and primary OS tumour displayed progressive expansion of the tumour and concurrent reabsorption of the HN and rat femur cortex, leading to a potential burst or fracture due to cortical effacement. Together, this suggests a mixed osteoblastic/osteoclastic disease phenotype which is characteristic for OS^36–38^.

Within the context of the model reported herein, osteoclastic-driven bone reabsorption was observed using Cathepsin K and TRAP activity as established markers for osteoclasts^39^, at both the interface between the OS tumour and the rat femur, and at various sites throughout the tumour, away from the rat femur. In alignment with the clinical scenario, most OS tumours are both osteoblastic and osteolytic^40^, yet the role of osteoclasts in OS progression is controversial. Some studies observed that OS cells increase osteoclast production and osteoclastic activity, for example through the interaction of OS-derived receptor activator of nuclear factor-KB ligand (RANKL), leading to pathological bone destruction and osteolytic lesions^41–43^. Increased osteoclastic activity leads to bone destruction and release of growth factors such as insulin-like growth factor I (IGF-I) and transforming growth factor β (TGF-β), thereby promoting OS growth^44^. In contrast, loss of osteoclasts or osteoclast inhibition with zoledronic acid has been associated with disease progression and formation of lung metastases^39,45^. In this study, we observed lung metastases in 100% of the rats, in conjunction with up-regulation of osteoclastic activity compared to normal bone within the primary tumour. Further studies are required to understand the relationship between osteoclastic activity and the development of OS lung metastases as a potential marker for disease severity, or for targeting new therapeutic interventions.

Histological and IHC analysis of the primary and metastatic OS tumours in our study revealed features distinctive to OS, such as osteoid-rich extracellular matrix (ECM) and evidence of ossification centres. The tumour macrophage population was investigated using rat pan-macrophage marker, CD68^46^. CD68^+^ staining was prominent throughout the TME, particularly at the tumour-bone interface and the tumour periphery. CD68^+^ macrophages are reported to be the most predominant tumour infiltrating leukocyte^47^, with a majority being the M2 phenotype^48,49^. Although both M1 and M2 tumour associated macrophage (TAM) phenotypes have been reported to contribute to OS tumour growth^50^, Shao et al. showed that M2 polarized TAMs enhanced OS initiation and stemness, associated with more aggressive disease^49^. This was supported by Zhou et al., who further noted that M2 TAMs drive tumour behaviour, including progression and metastasis^51^. To assess M2 polarized TAMs, we used IHC to detect CD163^+^ in the primary tumour. We observed CD163^+^ M2 TAMs only at the primary tumour periphery, with minimal infiltration into the tumour, suggesting that the CD68^+^ cells within the OS tumours were non-M2 myeloid lineage cell types, such as M1 TAM or osteoclasts, while M2 TAM congregate at the tumour invasive edge. We observed with label-free SHG imaging that the primary OS tumour edge was surrounded by a thick Col-I band, which potentially did not allow invasion of CD163^+^ to penetrate the OS tumour. The tumour ECM is known to act as a physical barrier to immune cell infiltration into the TME^52^. At the tumour edge, M2 macrophages play a crucial role in ECM remodelling, pro-migratory growth factor signalling and establishing an immunosuppressive TME^53^. Irrespective of polarization status, TAM infiltration into OS tumours has been associated with resistance to chemotherapy. In particular, neoadjuvant cisplatin therapy can stimulate TAMs to secrete larger quantities of IL-1β, thereby reducing OS cell sensitivity to cisplatin and leading to chemotherapy resistance^54^. Thus, TAMs have been proposed as a target for overcoming chemotherapy resistance^55^.

Although chemotherapy treatment can influence TAM populations, leading to the development of chemoresistance, chemotherapy treatment has also been reported to modulate tumour immune populations leading to synergistic cytotoxic and immunotoxic-mediated anti-cancer effects. Doxorubicin (DOX), a commonly used OS chemotherapy agent, has been reported to increase cancer cell immunogenicity and decrease immunosuppressive immune cell populations within colorectal cancers^56^. Doxorubicin can enhance tumour specific CD8^+^ T cell proliferation and reduces intra-tumoural myeloid derived suppressor cell populations contributing to the anti-tumour immune activity of chemotherapy treatment^57^. Clinically, primary OS tissue has high levels of infiltrating CD163^+^ M2 TAM and low levels of T cells^58^. Despite clear TAM presence within the primary OS tumour, a limitation within this model is a lack of endogenous B and T immune cells, which have important implications in OS disease progression, metastatic spread and response to chemotherapy^59–61^. In future work, we will reconstitute a humanized immune system within the immunocompromised rat model by human CD34^+^ haematopoietic stem cell transplantations^14,24^. This will allow to study the full spectrum of immune cell populations infiltrating the primary OS tumour and their impact on disease progression. Furthermore, as osteoclasts are derived from the myeloid lineage, this approach may also facilitate the development of human osteoclasts within the model, allowing the assessment of human osteoclast-driven bone and tumour microenvironment remodelling.

Despite the success of mouse models in achieving consistent lung metastasis rates in OS, few rat models have been able to replicate this rate in xenograft models, most likely due to the lack of highly immunocompromised rat models until recently^62^. Much of the rat OS research has centred around syngeneic models, such as Yu et al.. who developed a highly effective OS model using intra-femoral orthotopic injection of UMR106 cells into Sprague-Dawley (SD) rats, resulting in a 100% tumour take rate and a 93% lung metastasis rate^63^. Recent work from our team to create a humanized OS rat model resulted in 100% tumour take rate and 75% rats with lung metastatic tumour development^23^. Using a more highly immunocompromised rat model, we improved this metastatic take rate to 100% and could further observe that lung metastases were established 6-weeks after tumour initiation. Similar to our data, lung metastases in patients are detected as solid, calcified nodules with bilateral distribution^64^. We further observed that OS lung nodules were characterised by a proliferative periphery and a calcified osteoid centre. The lung microenvironment has a distinct oxygen partial pressure compared to bone tissue^65^. However, when lung tissue contains an osteoid centre, it becomes more comparable to bone. Both osteosarcoma (OS) tumours and lung nodules have a hypoxic microenvironment, which induces levels of Hypoxia-Inducible Factors, HIF-1α and HIF-2α within the tumour cells. These factors protect the OS and enable its survival^20,66^. Furthermore, the hypoxic microenvironment in the bone tumour microenvironment (TME) involves HIF-1α and CXCR4 pathways. The binding of Hypoxia-Responsive Elements (HRE) leads to the upregulation of the CXCR4 pathway, which promotes tumour development, migration, survival, and proliferation, and is associated with lung metastasis in clinical specimens^66,67^.

## Limitations

In this study, we did not include a direct control group in which OS cells were injected into the unmodified rat femur. This limits the direct comparisons of tumour growth and metastatic potential between the humanized and native bone environments. However, prior work in immunodeficient mouse models have demonstrated improved engraftment and more clinically relevant tumour phenotypes in humanized bone compared to murine bone, supporting our rationale for this approach^21^. Secondly, only the SaOS-2 cell line was used in this study. Although SaOS-2 forms bone and is well-suited to imaging-based analyses, it is a less aggressive and less metastatic cell line than other available lines, such as MG-63^68^. We chose the SaOS-2 line based on our previous extensive work with the model^13,20,23^. Importantly, SaOS-2 exhibits a more mature osteogenic phenotype compared to U2OS and MG-63 cell lines^69^, producing abundant osteoid and bone-like ECM in vivo, better recapitulating the bone-forming nature of human OS tumours. The slower growth kinetics and predictable progression also enable longitudinal assessment of tumour-bone interactions and metastatic spread in this study. Nonetheless, future studies will investigate the interaction of the humanized bone niche with an increased variety of cell lines and also patient-derived xenografts to improve translational potential. Finally, the immunocompromised rat model lacks endogenous B and T cells^62,70^, precluding analysis of tumour-adaptive immune interactions that influence disease progression and treatment response. Future studies incorporating a humanized immune system into the bone niche^24^ will assist with understanding tumour-immune-bone responses.

## Conclusion

We established a reproducible and highly metastatic humanized orthotopic OS model in immunocompromised rats that recapitulates key features of human disease, including mixed osteoblastic/osteoclastic bone remodelling and spontaneous lung metastasis in 100% of animals. The larger anatomical scale and longer lifespan of the rat offer advantages over mouse models, enabling longitudinal disease tracking and future integration of surgical or implant-based interventions^71,72^. Importantly, this model supports clinically relevant features such as osteoid formation within metastatic lung nodules and tumour progression from primary to secondary sites. Here, we provide a valuable platform which can also be used in the future to investigate OS biology, ECM-immune-tumour interactions, and response to therapeutic interventions. This platform lays the foundation for future studies to incorporate OS-PDX and humanized immune system reconstitution to improve translational relevance and support the development of more effective OS treatments.

## Supplementary Information

Below is the link to the electronic supplementary material.

Supplementary Material 1

## Data Availability

The datasets generated during and analysed during the current study are available from the corresponding author on reasonable request.
