# Supplementary material for: Dynamic assessment of a humanized bone tumour microenvironment reveals insights into osteosarcoma primary tumour remodelling and lung metastases
Source: Sci Rep. 2025 Nov 28;15:45619. doi: 10.1038/s41598-025-29941-z (PMC12753712; doi:10.1038/s41598-025-29941-z)
Supplement: Supplementary file 1 — Supplementary Material 1 [file 41598_2025_29941_MOESM1_ESM.docx]

**Dynamic assessment of a humanized bone tumour microenvironment reveals insights into osteosarcoma primary tumour remodelling and lung metastases**

**
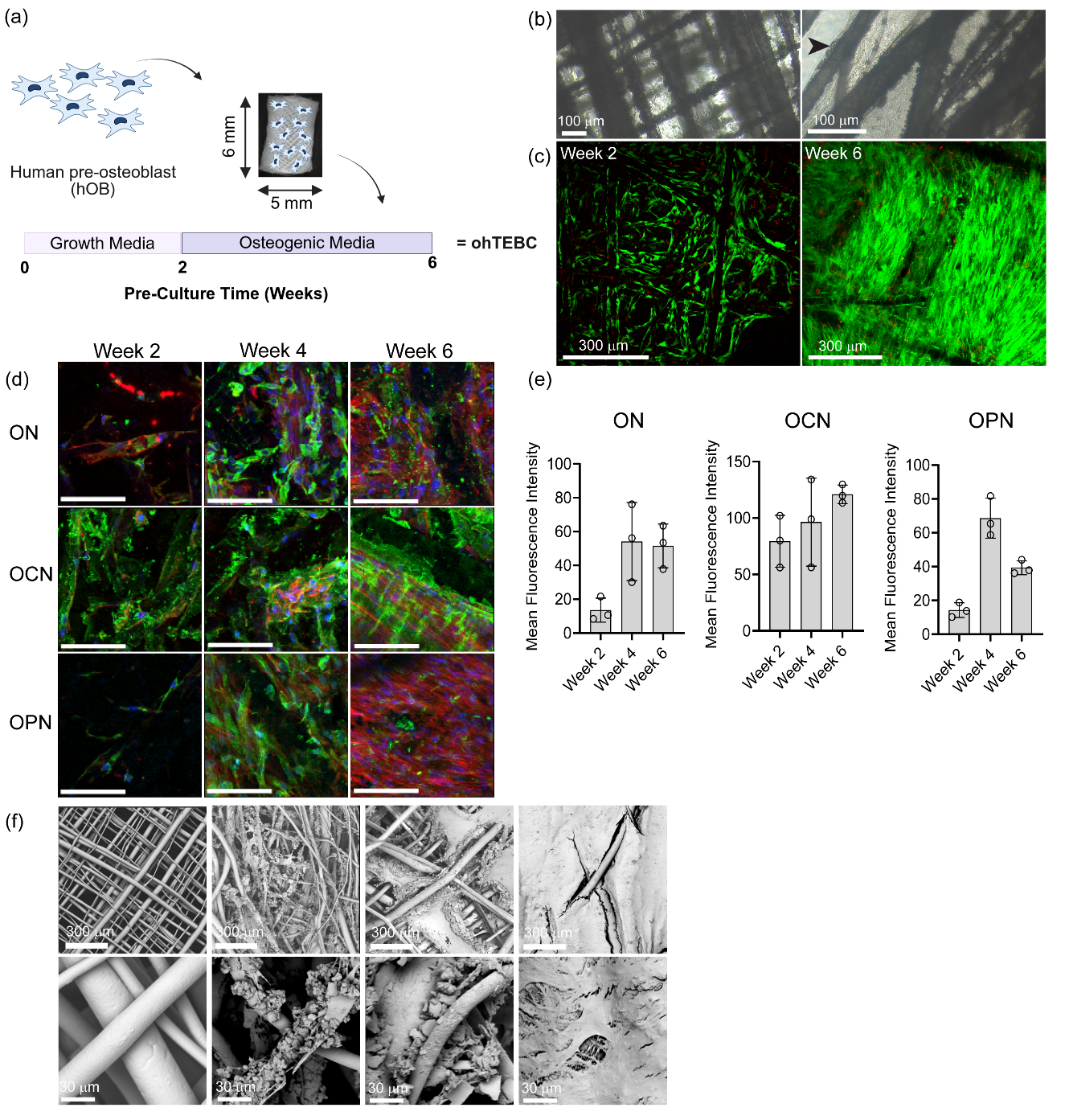
**

**Supplementary Figure 1. Creation and characterization of an *in vitro* orthotopic humanized tissue engineered bone construct (ohTEBC).**

**(a)** Schematic representation of the ohTEBC creation procedure. Human pre-osteoblasts (hOB) isolated from hip arthroplasty surgery were seeded onto melt electro-written (MEW) and calcium phosphate coated (CaP) medical-grade polycaprolactone (mPCL) scaffolds and cultured with growth media for 2 weeks, before switching to osteogenic culture media for a further 4 weeks to create the ohTEBC. **(b)** Brightfield microscope images prior to *in vivo* implantation show hOBs attached to the mPCL fibres, forming a continuous monolayer (asterisk) covering the mPCL-CaP fibres (arrowhead). **(c)** Live (green)/dead (red) confocal imaging performed on ohTEBC after 2 weeks and 6 weeks of *in vitro* culture demonstrates the coverage of viable hOBs on the ohTEBC over time. (**d)** Immunofluorescence of osteonectin (ON), osteopontin (OPN) and osteocalcin (OCN) confirms the expression of bone-related markers at weeks 2, 4 and 6 in the ohTEBC. Primary Antibody (green)/Actin (red). **(e)** Quantification of mean fluorescence intensity (MFI) of ON, OCN and OPN at each timepoint. (n=3 representative regions of interest) show ON and OPN peaked at week 2 whilst OCN steadily increased over time. **(f)** Scanning electron microscopy of mPCL scaffolds prior to CaP coating and cell seeding, and images of increasing magnification taken 2, 4 and 6 weeks after CaP coating and hOB seeding.


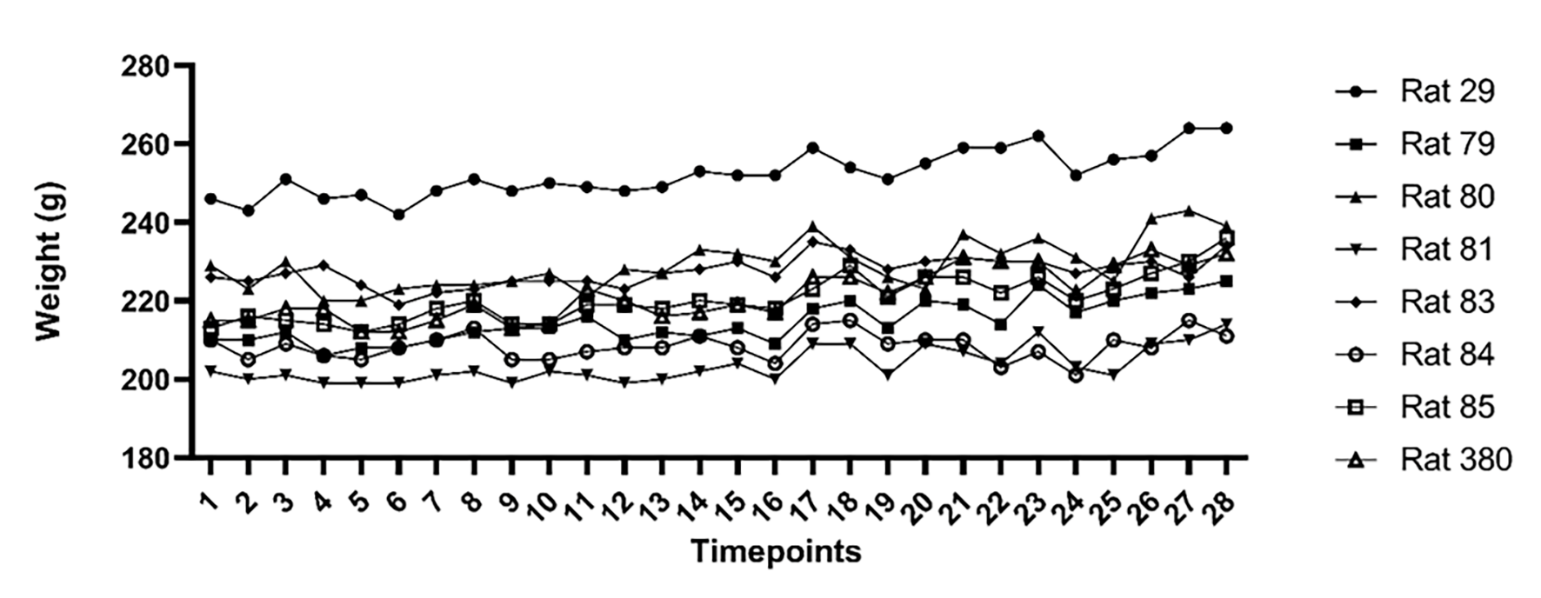


**Supplementary Figure 2. Rat weight is maintained for the study duration.**

Rats were weighed weekly and recorded (in grams). The rats maintained their weight over time.

**Supplementary Table. 1 Immunohistochemistry (IHC) Protocol Details.**

| Antigen | Raised in | Company | CatNo. [clone] | RRID | Antigen Retrieval | Dilution and incubation | DAB detection time | Description |
| --- | --- | --- | --- | --- | --- | --- | --- | --- |
| Ki-67* | Mouse | Agilent (Dako) | M724001-2  [MIB-1] | AB_2631211 | Tris-EDTA + 0.1% Tween-20, pH 9.0 (90 °C for 45 min) | 1:100, 1 h at RT | 3 min | Proliferation marker. |
| Lamin A + C * | Rabbit | Abcam | ab108595  [EPR4100] | AB_10866185 | Tris-EDTA + 0.1% Tween-20, pH 9.0 (95 °C for 5 min) | 1:300, 1 h at RT | 2 min | Human-specific cell marker. |
| Osteocalcin (OC) * | Mouse | Abcam | ab13418  [OC4-30] | AB_300332 | Proteinase K (RT for 15 min) | 1:300, 16 h (overnight) at 4 °C | 1 min | Osteoblast marker and a marker for bone formation. |
| Human specific Type I Collagen (hsCol-I)* | Rabbit | Abcam | ab138492  [EPR7785] | AB_2861258 | Tris-EDTA + 0.1% Tween-20, pH 9.0 (95 °C for 5 min) | 1:500, 1 h at RT | 1 min | Secreted protein that forms fibrillar collagen within the ECM. |
| Type II Collagen (Col – II) | Mouse | DSHB | II-II6B3 | AB_528165 | Proteinase K (RT for 15 min) | 1:200, 16 h (overnight) at 4 °C | 1 min | Protein found in articular and hyaline cartilage that detects presence of endochondral ossification |
| Cathepsin K | Mouse | Santa Cruz Biotechnology | sc-48353 | AB_2087687 | Citrate Buffer  (95 °C for 5 min) | 1:400, 1 h at RT | 30 s | Secreted by activated osteoclasts and degrades collagen and other matrix components during bone resorption. |
| CD68 | Rabbit | Abcam | ab125212 | AB_10975465 | Proteinase K (RT for 5 min) | 1:300, 1 h at RT | 15 s | Pan macrophage marker in rats. |
| CD163 | Rabbit | Abcam | ab182422 | AB_2753196 | No Antigen Retrieval | 1:200, 1 h at RT | 40 s | Selective M2 macrophage marker. |
| Alkaline Phosphatase | Mouse | Santa Cruz Biotechnology | Sc166261  TNAP (F-4) | AB_2226252 | Proteinase K (RT for 5 min) | 1:100, 1 h at RT | 2 min | Regulates bone mineralization. |
| Alpha Smooth Muscle Actin | Mouse | Abcam | ab7817 | AB_262054 | Proteinase K (RT for 10 min) | 1:500, 1 hr, at RT | 30 s | Marker for smooth muscle cells, cytoskeleton and fibrosis. |
| vWF | Rabbit | Agilent (Dako) | IR52761-2 | AB_2810304 | Proteinase K (RT for 5 min) | Ready to Use | 20 s | Vasculature marker. |
| Rabbit isotype control | Rabbit | ThermoFisher Scientific | 08-6199 | AB_2532942 | As per primary antibody | Neat | As per primary antibody | Non-immune serum IgG control. |
| Mouse isotype control | Mouse | ThermoFisher Scientific | 08-6599 | AB_2532952 | As per primary antibody | Neat | As per primary antibody | Non-immune serum IgG control |

*These antibodies react with human cells and tissues, but not with rat cells and tissues when used in immunohistochemistry.


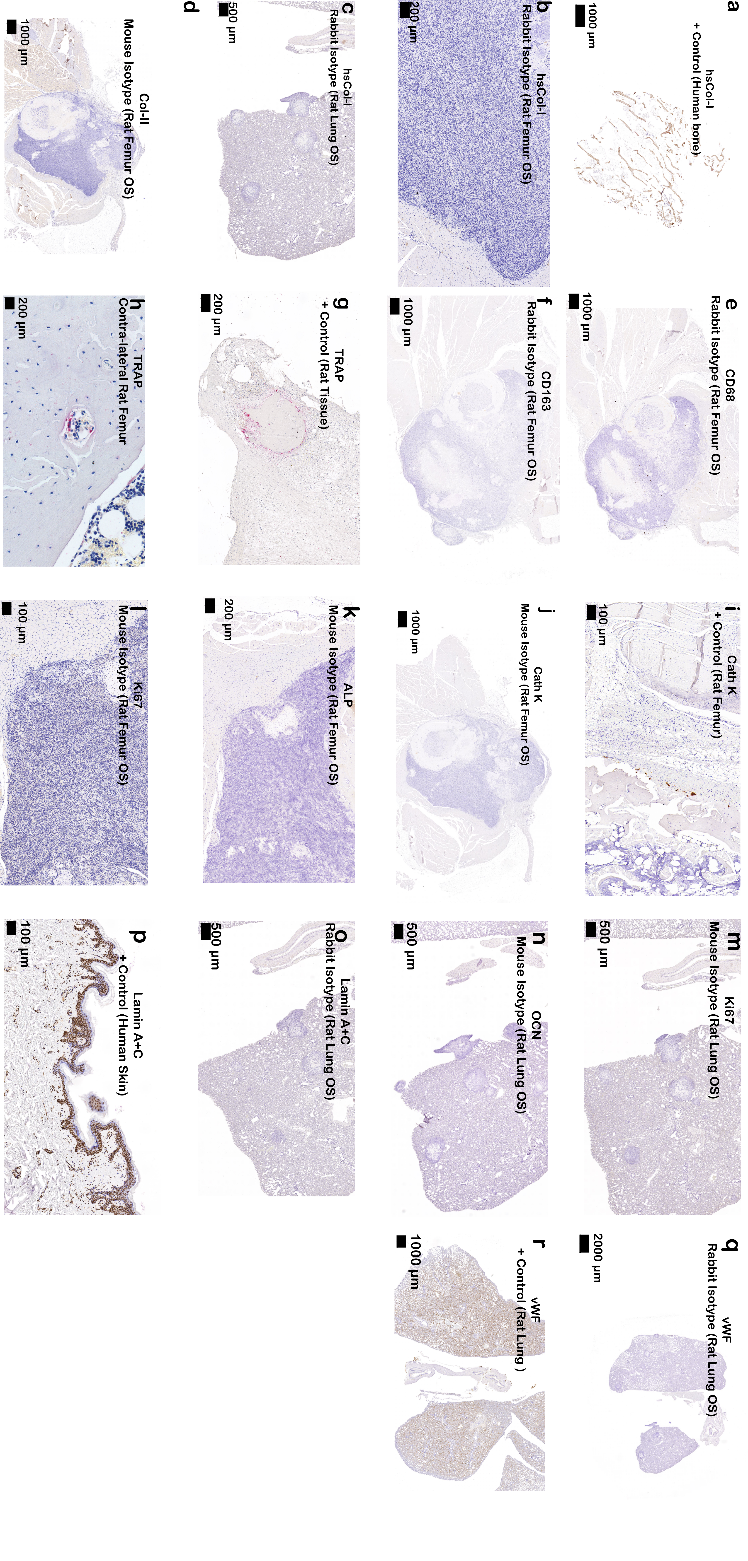


**Supplementary Figure 3. IHC Protocol isotype and positive controls.**

IHC evaluation of primary OS and lung metastasis with positive and negative isotype controls against **(a-c)** human specific collagen I (hsCol-I), **(d)** collagen II (Col-II), **(e)** cluster of differentiation 68 (CD68), **(f)** cluster of differentiation 163 (CD163), **(g-h)** tartrate-resistant acid phosphatase (TRAP), **(i-j)** Cathepsin K (Cath K), **(k)** alkaline phosphatase (ALP), **(l-m)** (Ki67), **(n)** osteocalcin (OCN), **(o-p)** Lamin A+C and **(q-r)** von Willebrand Factor (vWF).

**
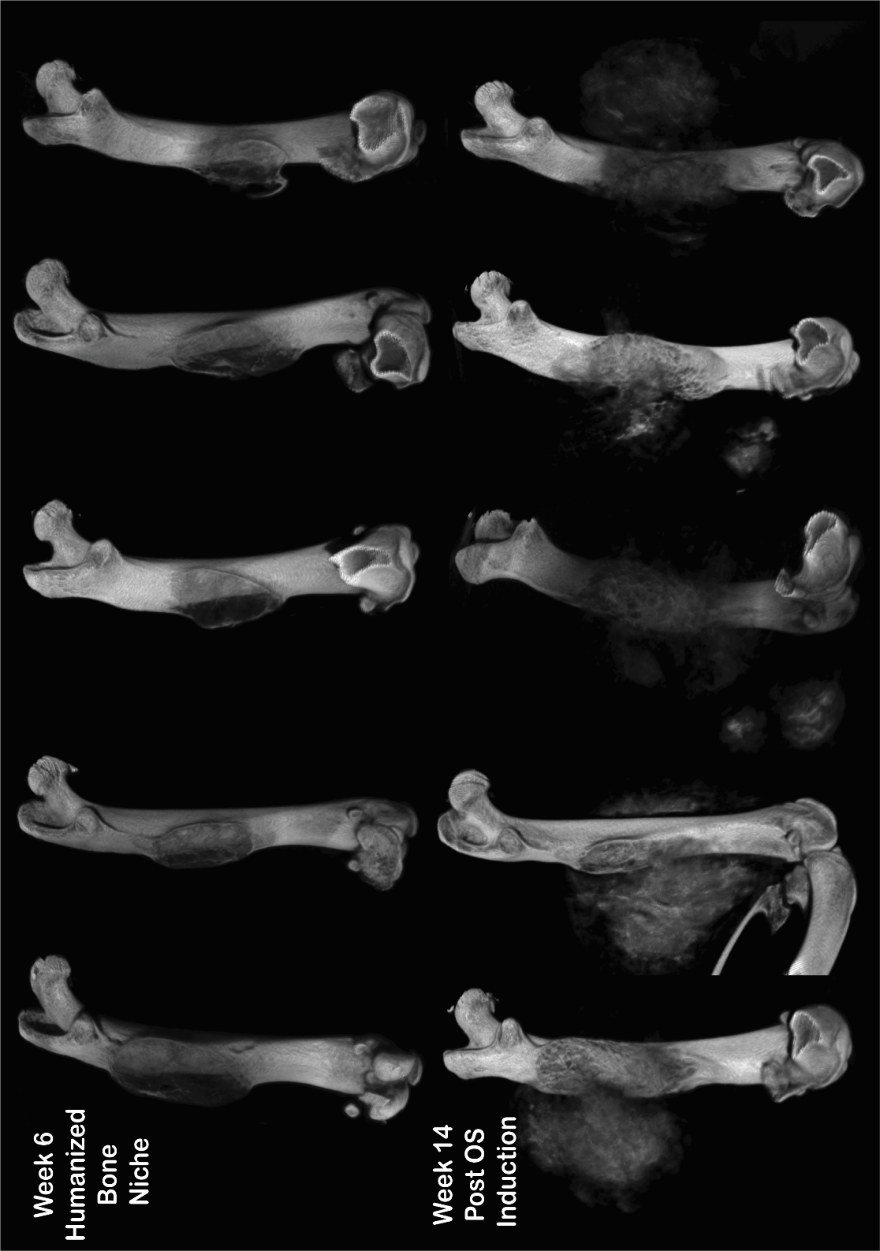
**

**Supplementary Figure 4. Orthotopic injection of SaOS-2-Luc cells into the humanized bone niche creates an orthotopic OS tumour**

Anaesthetised rats were placed into a Molecubes X-Cube unit and *in vivo* CT was performed. *In vivo* µCT reconstruction images comparing the morphology of the humanized bone niche 6 weeks after orthotopic ohTEBC implantation versus the orthotopic OS tumour 14-weeks after injection of SaOS-2-luc cells into the humanized bone niche (n=5 representative rats). µCT 3D volumes were created using Bruker CTan (CT Analyser Version 1.18.8.0 +) to crop the region of interest, CTvox Version 3.3.1 Bruker microCT was then used to create the 3D volume as visualised.


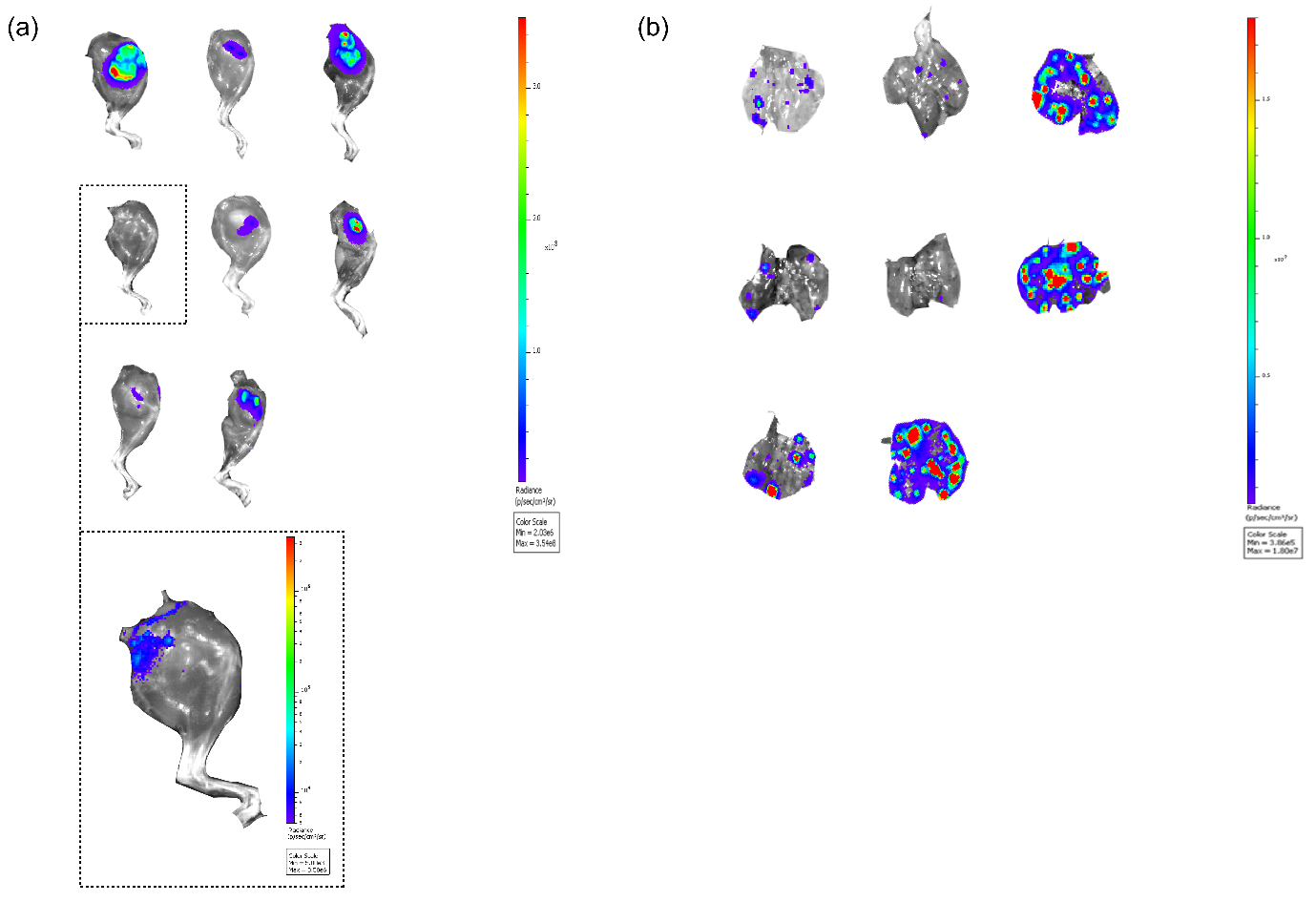


**Supplementary Figure 5. *Ex vivo* BLI images of OS tumour and lung tissues**

Rats were injected with luciferin prior to humane killing. Tissues (right leg and lung) were collected and placed inside an IVIS Spectrum (Perkin Elmer, USA) and images captured. **(a)** Post-mortem *ex vivo* BLI signal of the right femur shows presence of primary tumour in all rats. Inset panel of one rat with lower BLI signal is shown on an individual scale, confirming BLI signal. **(b)** Lung tissues were collected and analysed *ex vivo* via BLI imaging, confirming metastasis from primary tumour to the lungs in 100% of the rats. Living Image Version 4.8.2 was used to create the image.


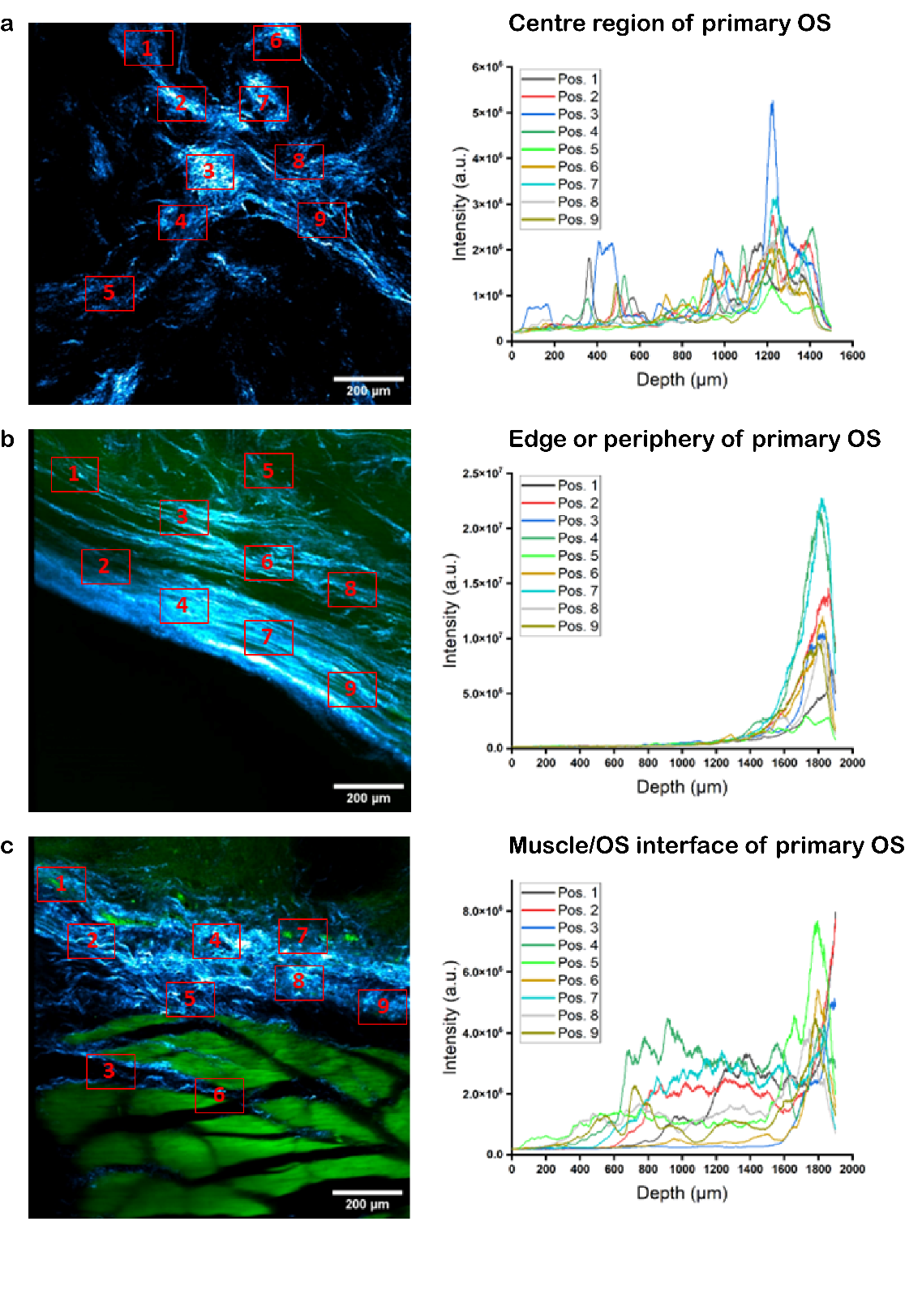


**Supplementary Figure 6. Second harmonic generation (SHG) imaging of OS tumour tissue to visualize tumour collagen networks.**

SHG imaging was performed to assess the rich Col-I ECM at **(a)** the centre of the primary OS tumour at the femur which displayed a dense star-like appearance and **(b)** the outer edge of the body of the OS tumour where there was increased intensity in zones 2, 4 7 and 9. **(c)** The OS/Muscle interface is shown and appears that Col-I is infiltrating between muscle fibres as evidenced by regions 3 and 6. (Blue: Collagen I fibres in SHG image). Units: Intensity (a.u).

**
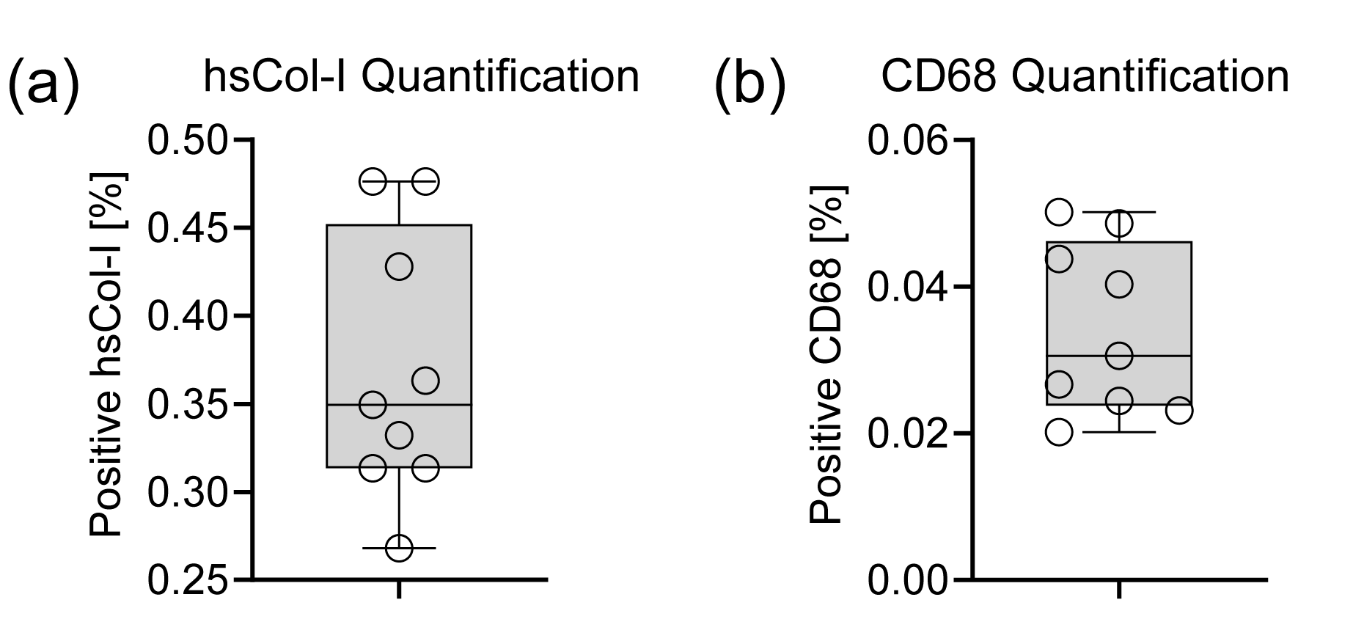
**

**Supplementary Figure 7. Immunohistochemistry quantification was performed at the primary OS tumour investigating hsCol-I and CD68 positive cells.**

IHC was performed to assess the rich hsCol-I ECM (36.89% ± 7.48% of primary tumour was positive via IHC) **(a)** within the primary OS tumour at the femur and **(b)** CD68 staining (3.423 ± 1.16% of primary tumour was positive via IHC) which is a pan-macrophage markers within rat.


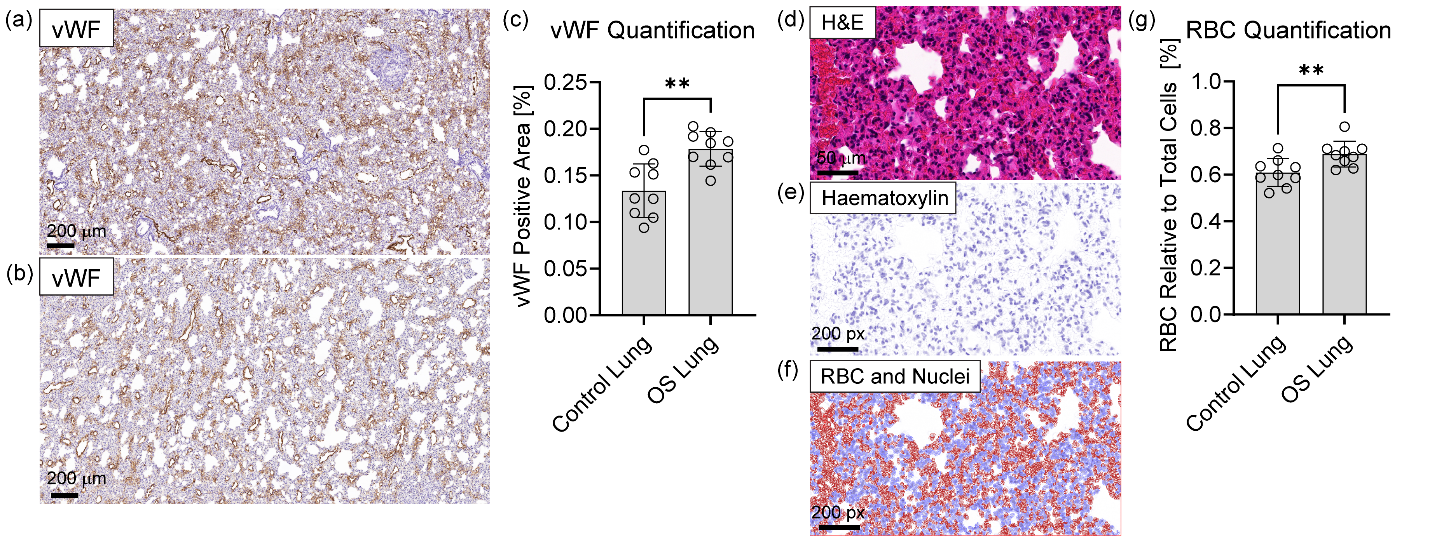


**Supplementary Figure 8. OS lung metastases increase vascular density and red blood cell infiltration into the lung microenvironment**

Lung tissue was stained for vWF using IHC in **(a)** OS and **(b)** normal rat lung tissue. **(c)** Quantification of vWF positive immunoreactivity revealed a significantly higher vWF positive area in lung tissue from metastatic OS tumours compared to normal tissue control (P = 0.0012). **(d)** H&E sections of lung tissue from metastatic OS and normal rat tissue were analysed using ImageJ. **(e)** haematoxylin staining was deconvoluted and **(f)** the RBC and nuclei separated. **(g)** Quantification of total RBC area revealed greater RBC positive area in lung tissue containing lung metastases compared to normal rat lung tissue (P = 0.0096). Data are represented as mean ± SD (n=9 ROI).
